# Calreticulin and JAK2V617F driver mutations induce distinct mitotic defects in myeloproliferative neoplasms

**DOI:** 10.1038/s41598-024-53240-8

**Published:** 2024-02-02

**Authors:** Kristin Holl, Nicolas Chatain, Susanne Krapp, Julian Baumeister, Tiago Maié, Sarah Schmitz, Anja Scheufen, Nathalie Brock, Steffen Koschmieder, Daniel Moreno-Andrés

**Affiliations:** 1 Institute of Biochemistry and Molecular Cell Biology, Faculty of Medicine, RWTH Aachen University, Aachen, Germany; 2 Department of Hematology, Oncology, Hemostaseology, and Stem Cell Transplantation, Faculty of Medicine, RWTH Aachen University, Aachen, Germany; 3 Center of Integrated Oncology Aachen Bonn Cologne Düsseldorf (CIO ABCD), Aachen, Germany; 4 Institute for Computational Genomics, Joint Research Center for Computational Biomedicine, Faculty of Medicine, RWTH Aachen University, Aachen, Germany

**Keywords:** Haematological diseases, Haematological cancer, Haematological cancer, Cell division, Chromosome segregation, Mitosis, Mechanisms of disease

## Abstract

Myeloproliferative neoplasms (MPNs) encompass a diverse group of hematologic disorders driven by mutations in JAK2, CALR, or MPL. The prevailing working model explaining how these driver mutations induce different disease phenotypes is based on the decisive influence of the cellular microenvironment and the acquisition of additional mutations. Here, we report increased levels of chromatin segregation errors in hematopoietic cells stably expressing CALRdel52 or JAK2V617F mutations. Our investigations employing murine 32D^MPL^ and human erythroleukemic TF-1^MPL^ cells demonstrate a link between CALRdel52 or JAK2V617F expression and a compromised spindle assembly checkpoint (SAC), a phenomenon contributing to error-prone mitosis. This defective SAC is associated with imbalances in the recruitment of SAC factors to mitotic kinetochores upon CALRdel52 or JAK2V617F expression. We show that JAK2 mutant CD34 + MPN patient-derived cells exhibit reduced expression of the master mitotic regulators PLK1, aurora kinase B, and PP2A catalytic subunit. Furthermore, the expression profile of mitotic regulators in CD34 + patient-derived cells allows to faithfully distinguish patients from healthy controls, as well as to differentiate primary and secondary myelofibrosis from essential thrombocythemia and polycythemia vera. Altogether, our data suggest alterations in mitotic regulation as a potential driver in the pathogenesis in MPN.

## Introduction

Philadelphia chromosome-negative myeloproliferative neoplasms (Ph-neg. MPNs) are a heterogeneous group of clonal hematopoietic disorders clinically subdivided into polycythemia vera (PV), essential thrombocythemia (ET), and primary myelofibrosis (PMF)^1^. The mutations in the genes of the Janus kinase 2 (JAK2), calreticulin (CALR), or the thrombopoietin receptor (TPOR/MPL) are driver mutations of these diseases^2,3^. Their occurrence and variant allele frequency, together with specific bystander mutations, determine the clinical features, disease severity, and whether these diseases evolve with dismal prognosis and decreased survival^3–6^, towards secondary myelofibrosis (SMF), secondary acute myeloid leukemia and/or secondary solid tumors^7,8^.

The main pathogenic molecular signaling event of Ph-neg. MPNs is the constitutive activation of JAK2-STAT-dependent signaling pathways by mutations in CALR, JAK2, or the MPL receptor^2,3^. Yet, non-canonical mechanisms of mutant JAK2^9^, and CALR^10^ have been recently linked to aspects of the disease pathology. However, the molecular mechanisms of the phase transition towards acute disease states are poorly defined^2,5^.

In contrast to other myeloid neoplasms such as primary acute myeloid leukemia (AML)^11,12^, myelodysplastic syndromes^13,14^, or chronic myeloid leukemia^15,16^, the cytology and molecular status of mitosis in Ph-neg. MPNs have not been studied in detail. However, karyotype abnormalities likely caused by chromatin segregation defects due to flawed mitosis are present in up to 5% of ET, 20% of PV, and 57% of PMF cases at the time of diagnosis^4,17,18^ and accumulate over time, especially at blast-phase transformation^19–22^ and frequently are associated with unfavorable prognosis and decreased survival^23–25^. Therefore, mitotic defects induced by driver Ph-neg. MPN mutations could play a role in the pathological mechanisms and contribute to the phase transition.

Mitosis is tightly regulated by the crosstalk between the kinases Aurora B, CDK1 (cyclin- dependent kinase 1)-Cyclin B1, and Polo-Like Kinase 1 (PLK1), and the protein phosphatase PP2A, as well as by the spindle assembly checkpoint (SAC)^26,27^. The latter constitutes a protein network recruited to chromosome kinetochores to ensure proper chromosome-spindle attachments and accurate chromatin segregation. It includes several evolutionarily conserved proteins, like BubR1, Aurora B, MAD1, MAD2, MPS1, CDC20, and kinesin motor proteins, such as CENP-E, which are required for precise SAC function^26,28^. Precise maintenance of the molecular equilibrium in gene expression and accurate subcellular positioning of these mitotic regulators play a critical role in preserving chromosome integrity and ensuring the stability of the karyotype^16,28–30^. Consequently, defects in mitotic regulation promote chromosome instability (CIN), acquisition and evolution of heterogeneous karyotypes, inflammation, and epigenetic dysregulation. All these pathological mechanisms are linked to the malignant transformation in many solid cancers^31–33^. Similarly, hematological malignancies^16^ such as AML^11,12^ and myelodysplastic syndromes^13,14^, show defects or dysregulation in crucial mitotic factors linked to CIN and heterogeneous karyotypes.

Here, we have analyzed the mitotic cytology in murine and human cells stably expressing CALRdel52 or JAK2V617F and found error-prone mitosis. The examination of the molecular status of key mitotic regulators suggests defective SAC function. Also, CD34 + Ph-neg. MPN patient cells display differential expression profiles of a subset of important mitotic regulators, including the SAC components BUB1, MAD2L1, INCENP, CDC20, CDK1, PLK1, and Aurora A/B.

## Results

### CALRdel52 and JAK2V617F 32D^MPL^ cells have a stress-sensitive and error-prone mitosis

**Figure 1:**
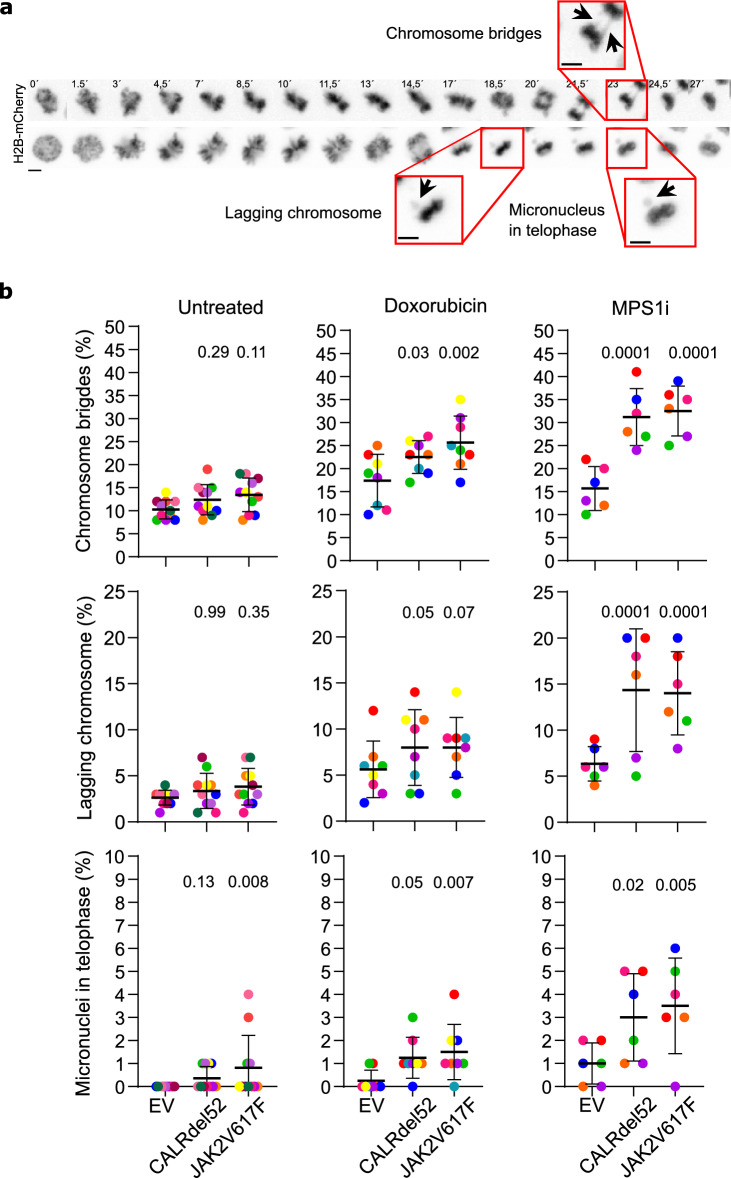
Chromatin segregation defects in murine CALRdel52 or JAK2V617F 32D cells. (**a**) Example of live-cell image galleries of mitotic JAK2V617F transduced 32D^MPL^ cells with chromatin bridges, lagging chromosomes, or micronuclei (insets). Scale bars 5 µm. (**b**) Plots showing the percentage of chromatin bridges, lagging chromosomes and micronuclei in untreated control (EV), CALRdel52 or JAK2V617F transduced 32D^MPL^ cells, or cells treated with 200 nM doxorubicin or 3 µM MPS1 inhibitor (MPS1i) NMS-P715 (6 to 11 independent color-coded experiments with 100 cells per experiment and condition, the horizontal black lines and dispersion bars indicate means and SD of the means). The significance *p*-values were obtained with the exact Fisher test (EV control versus mutant cells).

To investigate chromatin segregation and the duration of mitosis, we performed long-term live-cell imaging of murine 32D^MPL^ cells (Fig. 1a) for a duration of 20 h followed by image analysis. To exert its oncogenic capacity, mutant CALR is dependent on the expression of and interaction with the thrombopoietin receptor, MPL^34,35^. Therefore, to compare the mitotic phenotypes in similar growth and transduction conditions, all cell lines were transduced cells with the MPL receptor and were cultured in a medium supplemented with IL-3. In comparison with control 32D^MPL^ (EV) cells, 32D^MPL^ cells transduced with CALRdel52 or JAK2V617F showed a slight and non-significant increase in the numbers of chromatin bridges and lagging chromosomes (Fig. 1b). In contrast, the percentage of telophase micronuclei is significantly increased in the JAK2V617F mutant cell (*p* < 0.008, Fisher´s exact test). The occurrence of all three kinds of chromatin segregation errors further increases significantly in comparison to EV when DNA damage is induced with the chemotherapeutic agent doxorubicin^36^ (*p* < 0.05, Fisher´s exact test), or SAC malfunction with the antimitotic drug NMS-P715 (MPS1i)^37^ (*p* < 0.02, Fisher´s exact test), which inhibits the checkpoint kinase MPS1 (Fig. 1b). The average mitotic timing in untreated cells or upon treatment with doxorubicin or MPS1 inhibitor is similar between mutants and control (EV) transfected cells (Supplementary Fig. S1a). As expected, the treatment with the SAC inhibitor NMS-P715 reduced the mitotic timing with respect to untreated samples (Supplementary Fig. S1S1a). These data suggest that mitosis in CALRdel52 and JAK2V617F mutant 32D^MPL^ cells is stress-sensitive.

To determine whether inhibition of JAK2 signaling could ameliorate/overcome the mitotic phenotypes induced by the antimitotic drug NMS-P715, we treated the cells with ruxolitinib for 4 h, a selective JAK1 and JAK2 inhibitor widely used for the treatment of Ph-neg. MPNs^38,39^, before NMS-P715 was added. 32D^MPL^ cells transduced with CALRdel52 or JAK2V617F but not control 32D^MPL^ (EV) cells showed significantly decreased (*p* < 0.05, Fisher´s exact test) numbers of chromatin bridges when treated with ruxolitinib (Supplementary Fig. S1b). In the presence of ruxolitinib, the numbers of lagging chromosomes and telophase micronuclei were significantly decreased (*p* < 0.05, Fisher´s exact test) in control 32D^MPL^ (EV) cells as well as in CALRdel52 and JAK2V617F mutant 32D^MPL^ cells (Supplementary Fig. S1b). After treatment with ruxolitinib, the level of all mitotic errors in CALRdel52 and JAK2V617F mutant 32D^MPL^ cells was comparable to DMSO-treated control 32D^MPL^ (EV) cells. In contrast to DMSO-treated cells, ruxolitinib-treated cells had significantly elongated mitotic timing (*p* < 0.05, One-Way ANOVA) (Supplementary Fig. S1c). These data suggest that JAK2 inhibition with ruxolitinib generally protects the cells against the mitotic defects induced by SAC malfunction, albeit this was more prominent in CALRdel52 and JAK2V617F mutant cells.

### A weakened SAC contributes to error-prone mitosis in murine 32D^MPL^ CALRdel52 and JAK2V617F cells

JAK2V617F^9,40^ and CALRdel52^41^ mutations have been linked to increased ROS production. In addition, JAK2V617F is also linked to replication stress^42^, and to lower p53 levels, a factor which is critical for the DNA damage response^43^. Replication stress and DNA damage signaling pathways, together with mitotic dysregulation, are well-known sources of karyotype aberrations such as aneuploidy, CIN, and genomic instability^44^. Therefore, we investigated whether the observed error-prone mitosis in CALRdel52 and JAK2V617F cells after doxorubicin treatment could be due to an altered response to DNA damage or replication stress.

First, we tested whether CALRdel52 or JAK2V617F transduced 32D^MPL^ cells show increased levels of double-strand breaks during entry into mitosis as compared to control (EV) cells by immunofluorescence staining of γ-H2AX (H2AX S139ph), a well-described marker for DNA damage^45^. Visual inspection of γ-H2AX foci in untreated prometaphase EV and mutant cells revealed similar low levels of DNA damage. As expected, the number of γ-H2AX foci increased to a similar extent in control (EV) and mutant cells after doxorubicin treatment (Supplementary Fig. S2a). In agreement with the literature^43^, p53 basal levels in untreated JAK2V617F cells were much lower than in control EV cells or CALRdel52 (Supplementary Figs. S1b and S8). After doxorubicin treatment, p53 levels increased more than four-fold in all the cell lines. These results indicate that DNA damage is similarly induced by doxorubicin in control (EV) and mutant cells during mitotic entry and all cells showed a comparable functional stabilization of p53 after genotoxic stress.

**Figure 2:**
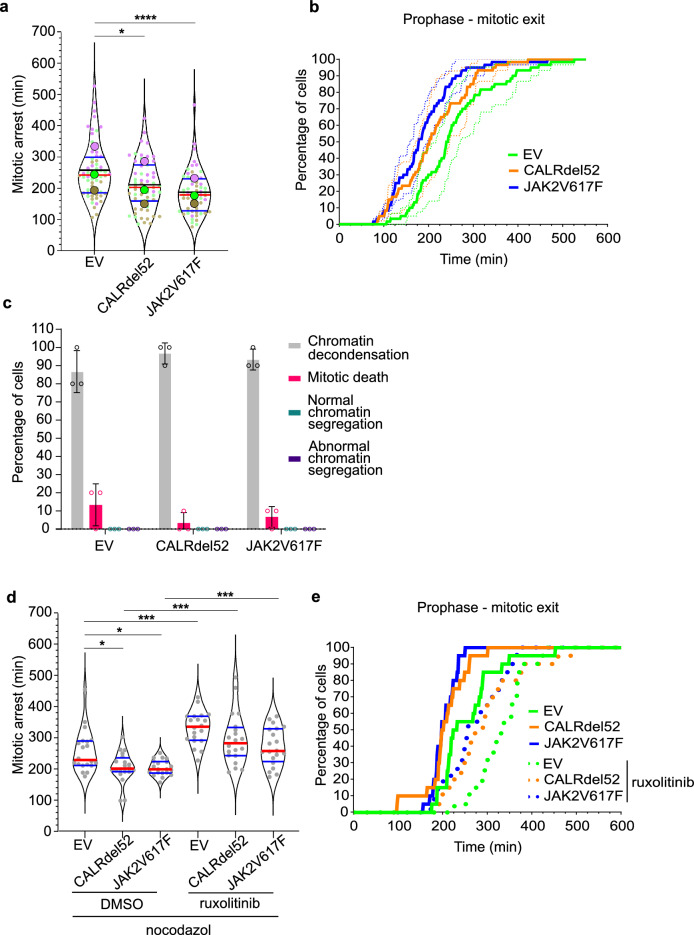
CALRdel52 and JAK2V617F mutations weaken the spindle assembly checkpoint. (**a**) Duration of mitotic arrest in 100 ng/ml nocodazole treated control (EV), CALRdel52 and JAK2V617F 32D^MPL^ cells co-expressing H2B-mCherry. The violin superplots show pooled data from three independent (color-coded) experiments with 20 cells per experiment and cell line. The thick colored dots indicate independent experiment means. Horizontal red lines indicate medians, black lines mean, and the blue lines quartiles of the pools. One-Way ANOVA (Kruskal–Wallis with Dunn’s post-test; *p < 0.05; ****p < 0.001). (**b**) Plots show the fraction of cells that exit mitosis at the given time after mitotic entry in the presence of 100 ng/ml nocodazole. Bold lines indicate mean, and SDs are indicated by dotted lines. (**c**) The outcome of the nocodazole-induced mitotic arrest was determined in control (EV) or CALRdel52- or JAK2V617F transduced 32D^MPL^ cells. Cell fates were categorized into four groups: spindle-less mitotic exit with direct chromatin decondensation (grey), cell death during mitotic arrest (red), normal chromatin segregation (green), and abnormal chromatin segregation (violet). Columns indicate the means of three independent experiments with 10 cells each, error bars SDs, and points the individual data points. No differences were found between the control (EV) to both mutants within each category. Two-Way ANOVA with Dunnet post-test. (**d**) Duration of mitotic arrest induced with 100 ng/ml nocodazole in the presence or absence of 1 µM ruxolitinib for 4 h in control (EV), CALRdel52 and JAK2V617F 32D^MPL^ cells co-expressing H2B-mCherry. The violin blots show data from 20 random cells (grey dots) per cell line and treatment. Horizontal red lines indicate medians and blue lines quartiles. (One-Way ANOVA (Kruskal–Wallis with uncorrected Dunn’s post-test; *p < 0.05; ****p < 0.001). (**e**) Plots show the fraction of cells treated with 100 ng/ml nocodazole that exit mitosis at the given time after mitotic entry in absence (dotted lines) or presence (bold lines) of 1 µM ruxolitinib.

To test whether defects in SAC could contribute to the observed increase of chromatin segregation errors in CALRdel52 and JAK2V617F mutant cells, we challenged them with the spindle poison nocodazole. Cells with a weakened or defective SAC escape faster from the nocodazole-induced mitotic arrest^29,46^. Nocodazole treatment induced mitotic arrest in all three cell lines (Fig. 2a, b; Supplementary Fig. S1d). However, compared to control (EV) cells (257 ± 45 min), CALRdel52 (210 ± 36 min, *p* < 0.02 one-way ANOVA) or JAK2V617F (186 ± 35 min, *p* < 0.0001) transduced 32D^MPL^ cells showed significantly shorter mitotic arrest and faster mitotic exit (Fig. 2a, b; Supplementary Fig. S1d).

The outcomes of mitotic arrest induced with microtubule inhibitors are diverse among cancer and normal cell lines due to the different cellular pathways they induce^47,48^. The current ‘competing networks-threshold’ model proposes that cell fate determination of either cell death or extended survival hinges on which of the two thresholds is reached first: either the activation of pro-apoptotic caspases or the degradation of Cyclin B1 leading to mitotic slippage^47,49^. To discriminate between these two options, we directly analyzed the fate of individual cells under mitotic arrest. The fraction of cells with mitotic death after mitotic arrest upon nocodazole treatment is very low (< 10%) and without significant differences between control (EV), CALRdel52, and JAK2V617F cells (two-way ANOVA with Dunnet post-test) (Fig. 2c). In all conditions the arrested cells mostly escaped mitotic arrest by mitotic slippage without significant differences between the different cell types (two-way ANOVA with Dunnet post-test) (Fig. 2c).

**Figure 3:**
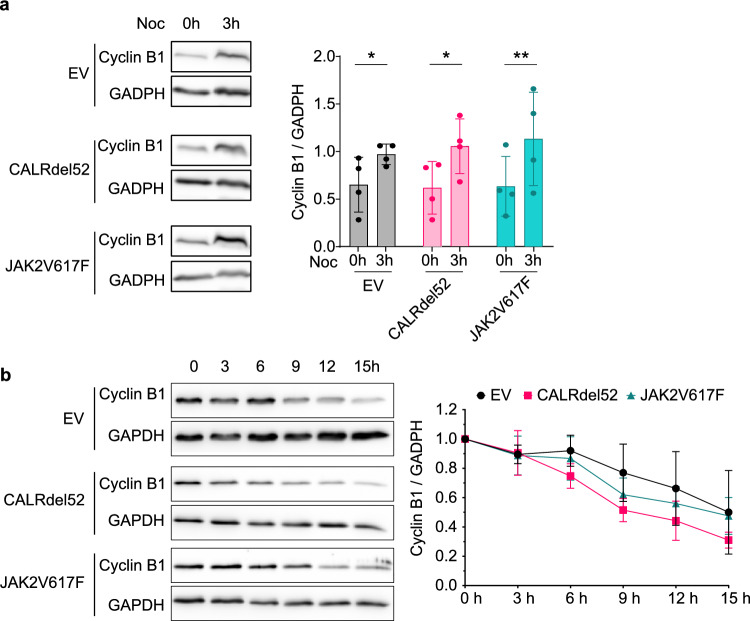
Cyclin B1 degradation is faster in cells expressing CALRdel52 and JAK2V617F mutations. (**a**) Cropped western blots of Cyclin B1 and GADPH as loading control from whole cell extracts without and with 200 ng/ml nocodazole treatment for 3 h. The left panel shows Cyclin B1 expression normalized to GAPDH. Columns indicate the means, error bars the SDs, and the dots the values of four independent experiments. Two-way ANOVA (Fishers LSD post-test; *p < 0.05; **p < 0.001). (**b**) Time course of Cyclin B1 degradation. Cells were arrested in mitosis for 3 h with nocodazole (200 ng/ml). The left panel shows the time course of Cyclin B1 expression normalized to GAPDH and 0 h. Data points indicate the mean; the error bars the SEM from three independent experiments. For statistical analysis, see Supplementary Fig. S3. Two-way ANOVA (Dunnett post-test; *p < 0.05; **p < 0.01; ***p < 0.001). For uncropped WB see Supplementary Fig. S6.

It has been proposed that murine cells are naturally more resistant than human cells to mitotic poisons due to the presence of clearance systems^50^. Therefore, we determined whether Cyclin B1 accumulation was different between control (EV) and CALRdel52 or JAK2V617F cells after 3 h of nocodazole treatment, which is the lower average time the cells (JAK2V617F cells, Fig. 2a) spend in mitotic arrest before they undergo slippage. Cyclin B1 accumulated similarly in all three cell lines (Fig. 3a). Once accumulated, Cyclin B1 was degraded faster in cells expressing CALRdel52 and JAK2V617F mutations (Fig. 3b; Supplementary Figs. S3 and S6) suggesting that a weakened SAC contributes to the error-prone mitosis in these cells.

To determine whether inhibition of JAK2 signaling was able to improve the SAC defects, we treated the cells with ruxolitinib for 4 h before challenging them with nocodazole. CALRdel52 (295 ± 80 min) or JAK2V617F (269 ± 60 min), as well as control 32D^MPL^ (EV) (330 ± 52 min) cells treated with ruxolitinib, showed extended mitotic arrest and slower mitotic exit (p < 0.05; Kruskal–Wallis test with uncorrected Dunn´s post-test) when compared with DMSO treated CALRdel52 (204 ± 48 min) or JAK2V617F (203 ± 24 min) as well as control 32D^MPL^ (EV) (256 ± 67 min) cells (Fig. 2d, e). The slower mitotic exit in 32D^MPL^ CALRdel52 or JAK2V617F cells was comparable to that in untreated control 32D^MPL^ (EV) cells (Fig. 2 e). These data, together with the previously observed reduction in mitotic errors after ruxolitinib treatment (Supplementary Fig. S1b), suggest that JAK2 inhibition with ruxolitinib rescues the SAC malfunction in CALRdel52 and JAK2V617F mutant cells.

In order to exert its oncogenic capacity, mutant CALR requires the TPO receptor MPL. Therefore, we tested whether acute overstimulation of control 32D^MPL^ (EV) cells with TPO impacts the SAC similar to the expression of the CALRdel52 mutant. For this, control 32D^MPL^ (EV) cells cultured for 4 h with different TPO concentrations of (0, 10, 50, and 100 ng/ml) were challenged with nocodazole. A fraction of the TPO-treated cells in all concentrations showed faster mitotic exit than untreated cells (Supplementary Fig. S2c). The average time under mitotic arrest for cells treated with 10 ng/ml TPO (297 ± 34 min) was significantly shorter than that of untreated cells (344 ± 66 min; *p* < 0.05; Kruskal–Wallis test with uncorrected Dunn´s post-test) (Supplementary Fig. S2d). However, compared to untreated cells, higher concentrations of TPO did not significantly shorten the mitotic arrest time (50 ng/ml, 331 ± 69 min; 100 ng/ml, 324 ± 100 min; Kruskal–Wallis test with uncorrected Dunn´s post-test) (Supplementary Fig. S2d). This suggests that the SAC defects are mainly driven by the expression of JAK2V617F and CALRdel52, although there might be a partial contribution from MPL receptor-dependent signaling.

### CALRdel52 and JAK2V617F 32D^MPL^ cells express normal levels of major SAC factors

**Figure 4:**
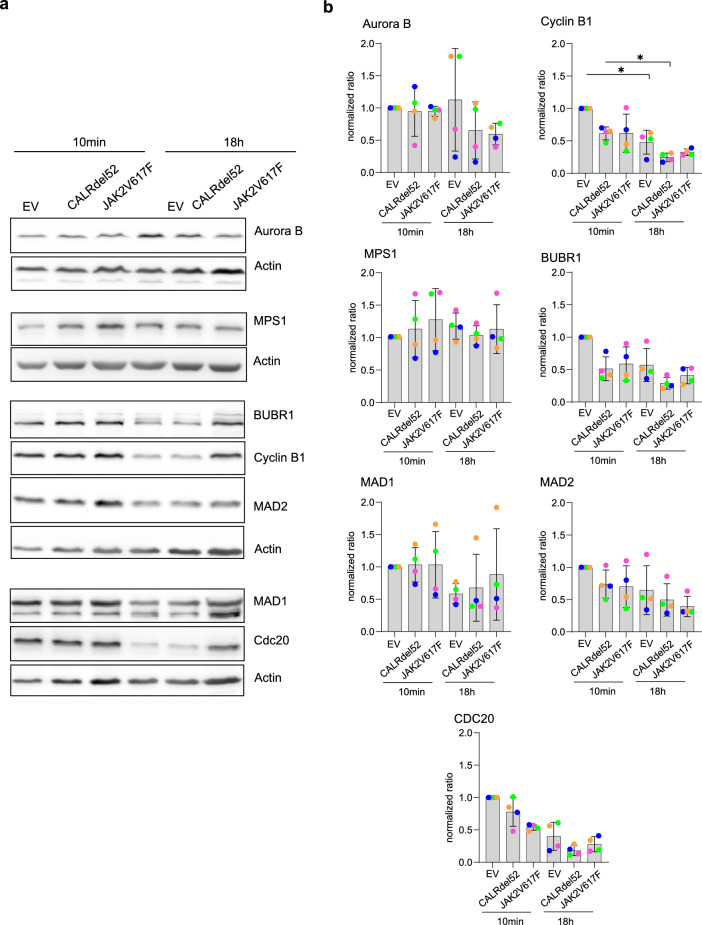
CALRdel52 and JAK2V617F mutations do not affect protein levels of several SAC factors. (**a**) Cropped western blots for the indicated SAC factors and the Actin as loading control from whole cell extracts generated from cells 10 min or 18 h after mitotic arrest induction with nocodazole (200 ng/ml). (**b**) Quantitative comparison of the expression of the indicated factors normalized to actin and EV control at t = 0 h. Grey columns indicate the mean, the error bars the SD, and the colored dots the values of four independent experiments. One-way ANOVA (Kruskal–Wallis test with Dunn´s post-test; *p < 0.05). For uncropped WB see Supplementary Fig. S7.

SAC factors are frequently downregulated in cancers, including AML^11,12,51^ and CML^15^. Therefore, we sought to determine whether defects in protein expression of several crucial SAC factors could be the cause of the observed mitotic defects in CALRdel52 or JAK2V617F 32D^MPL^ cells. We analyzed SAC protein levels through western blotting at approximately 10 min and 18 h after inducing spindle-less mitosis in the cells using nocodazole (Fig. 4a, b). Whole protein expression levels of key mitotic factors including aurora kinase B, MPS1, BubR1, MAD1, MAD2, and CDC20 did not change in CALRdel52 or JAK2V617F as compared to the corresponding control (EV) cells. As expected, Cyclin B1 levels decreased after 18 h of nocodazole arrest for all conditions with respect to the 10 min points (*p* < 0.05; Kruskal–Wallis test with Dunn´s post-test) (Fig. 4A–B). We also observed generalized but not significant decays in the levels of the other SAC factors (*p* < 0.05; Kruskal–Wallis test with Dunn´s post-test) at 18 h after nocodazole treatment such as for MPS1 and CDC20. The reason for this general reduction after long nocodazole arrest could be the rapid escape of 32D^MPL^ mouse cells from the nocodazole arrest under the experimental conditions (see Fig. 2b). In summary, these data suggest that CALRdel52 or JAK2V617F mutations affect SAC stability without impacting in the expression levels of the analyzed mitotic factors in murine myeloid cells.

### CALRdel52 and JAK2V617F 32D^MPL^ cells show defective kinetochore recruitment of SAC factors

**Figure 5:**
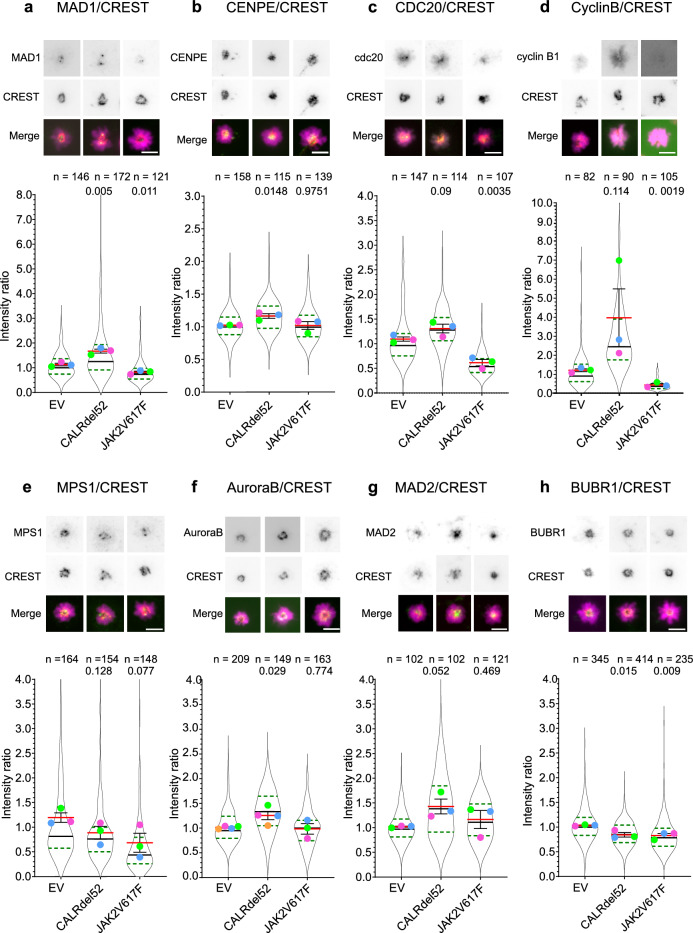
CALRdel52 and JAK2V617F mutations disturb kinetochore recruitment of SAC factors. Quantitative immunofluorescence for indicated SAC factors at kinetochores in 200 ng/ml nocodazole arrested control (EV), CALRdel52 or JAK2V617F transduced 32D^MPL^ cells stably expressing H2B-mCherry. (**a**)–(**h**) Representative immunofluorescences of SAC factors (green in overlay) and the kinetochore marker CREST (red in overlay). Violin superplots show the signal ratios of the different SAC factors to CREST at kinetochores as distribution of the pooled data from 3 or 4 independent experiments with n cells per condition as indicated. The horizontal black lines show medians, horizontal red lines the means, blue dotted lines quartiles, and dispersion bars the SDs of the independent experiments. The mean of each experiment is shown as a color-coded dot. The significance p-values were obtained with two-tailed t-test over the means of the independent experiments.

For proper SAC function, the relevant factors need to be recruited to unattached kinetochores. Accordingly, in addition to alterations of SAC factor expression levels, faulty localization or activity of SAC factors at kinetochores can cause SAC weakening^28–30,52,53^ and chromatin segregation errors. We thus analyzed kinetochore recruitment of SAC factors in nocodazole-arrested cells by quantitative immunofluorescence (Fig. 5a–g). CALRdel52 and JAK2V617F 32D^MPL^ cells showed altered intensities of several SAC factors at kinetochores (Fig. 5a–g). In CALRdel52 transduced cells the kinetochore localization of MAD1 (*p* < 0.01, two-tailed t-test), CENP-E (*p* < 0.05, two-tailed t-test), and aurora kinase B (*p* < 0.05, two tailed t-test) is increased while BubR1 (*p* < 0.05, two-tailed t-test) is decreased (Fig. 5a, b, f, h). In contrast, JAK2V617F mutants show reduced kinetochore localization of MAD1 (*p* < 0.05, two tailed t-test), CDC20 (*p* < 0.01, two-tailed t-test), Cyclin B1 (*p* < 0.01, two-tailed t-test), and BubR1 (*p* < 0.01, two-tailed t-test) (Fig. 5a, c, d, h).

In general, these recruitment unbalances agree with a weakened SAC. Overall, these imbalances affect several SAC factors. The effect of CALRdel52 transduction is opposite to that of JAK2V617F for the localization of MAD1, CDC20, and Cyclin B1, but shows the same tendency for BubR1 and MPS1, suggesting an alteration in the upstream regulatory network. We hypothesized that the kinetochore recruitment imbalances of these SAC factors could result from abnormal expression of master mitotic regulators like PLK1^54–57^ and/or PP2A^58–62^, which control mitotic kinetochore integrity and SAC signaling. However, we did not detect significant changes in the whole protein expression levels of either PLK1 or PP2A by Western blotting, which suggest that CALRdel52 or JAK2V617F mutations do not affect the expression of these master mitotic regulators in murine 32D cells (Supplementary Fig. S4a, b and S8).

### Transduction of human TF-1 cells with CALRdel52 and JAK2V617F results in SAC weakening

**Figure 6:**
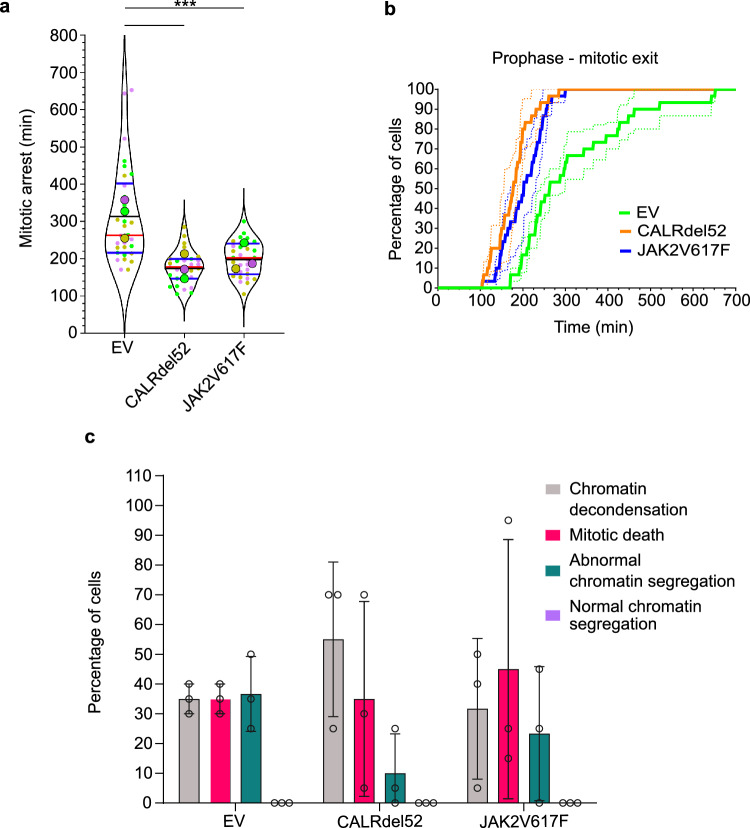
CALRdel52 and JAK2V617F mutations weaken the SAC in human TF-1^MPL^ cells. (**a**) Duration of mitotic arrest in 200 ng/ml nocodazole treated control (EV), CALRdel52 or JAK2V617F transduced TF-1^MPL^ cells co-expressing H2B-mCherry. The violin superplots show pooled data from three (color-coded) independent experiments with 10 cells per experiment and cell type. The thick colored dots indicate independent experiment means. Horizontal red lines indicate medians, black lines mean, and the blue lines are quartiles of the pool. One-Way ANOVA (Kruskal–Wallis with Dunn´s post-test; *p < 0.05 *p < 0.001). (**b**) Plots show the fraction of cells that escape from the mitotic arrest at the given time after mitotic entry in the presence of 100 ng/ml nocodazole. Bold lines indicate means, dotted lines the SDs. c) The outcome of the nocodazole-induced mitotic arrest was determined in control (EV), CALRdel52- or JAK2V617F transduced TF-1^MPL^ cells. Cells were categorized into four groups: spindle-less mitotic exit with direct chromatin decondensation (grey), cell death during mitotic arrest (red), normal chromatin segregation (green), and abnormal chromatin segregation (violet). Columns are the mean of three independent experiments with 10 cells each, individual experiments indicated, error bars SDs. No differences were found between control (EV) and CALRdel52- or JAK2V617F transduced cells in each category by Two-Way ANOVA with Dunnet post-test.

Subsequently, we questioned whether the identified mitotic abnormalities in murine cells stably expressing the driver mutations would manifest similarly in human cells. Therefore, we challenged the SAC in cytokine-dependent human erythroleukemic TF-1^MPL^ cells by nocodazole treatment. As 32D^MPL^ murine cells, TF-1^MPL^ cells showed significantly shorter mitotic arrest when transduced with CALRdel52 (177 ± 22 min, *p* < 0.0001, one-way ANOVA) or JAK2V617F (201 ± 23.5 min, *p* < 0.0001) compared to control (EV) cells (313 ± 75 min) (Fig. 6a, b). As compared to mouse 32D^MPL^ cells, a smaller fraction of TF-1^MPL^ cells exited mitotic arrest by mitotic slippage (Fig. 6c), in agreement with the observed higher sensitivity of human cells to mitotic poisons^48,50^. Here, no significant differences between control (EV), CALRdel52, and JAK2V617F TF-1^MPL^ cells upon arrest were observed (Two-Way ANOVA with Dunnet post-test). In addition, no significant differences in abnormal chromatin segregation after mitotic arrest induced with high nocodazole dosage between all the cell lines were detected (Fig. 6c). This has been also similarly observed by live-cell imaging in transformed cancer cell lines like HeLa or U-118 after long and high dosage treatment with nocodazole (100–300 nM)^48^. Together, these data suggest that CALRdel52 and JAK2V617F expression in human hematopoietic cells can perturb SAC functionally.

### Human HEL cells show error-prone mitosis with limited response to ruxolitinib

We then analyzed whether these mitotic errors and SAC defects were present in a different, non-cytokine-dependent human cell line. For this, human acute erythroid leukemia HEL cells, which naturally harbor the JAK2 V617F mutation homozygously, were treated for 4 h with DMSO or ruxolitinib and then incubated with and without the antimitotic drug NMS-P715 (MPS1i) to induce SAC malfunction. DMSO-treated HEL cells had misaligned chromosomes (6%), chromosome bridges (60%), and lagging chromosomes (14%) (Supplementary Fig. S4c, d), and these numbers remained unchanged after treatment with ruxolitinib (*p* > 0.05; Two-Way ANOVA). The treatment with the antimitotic drug NMS-P715 severely compromised the mitotic progression of HEL cells. NMS-P715 did not increase the number of chromosome bridges, but its severity, which impaired chromatin segregation in two daughter cells (77%) (*p* < 0.0001, Two-Way ANOVA) (Supplementary Fig. S4c), and the accurate quantification of chromosome bridges and lagging chromosomes. The treatment with NMS-P715 also significantly increased the number of misaligned chromosomes and micronuclei (*p* < 0.0001, Two-Way ANOVA). Under ruxolitinib treatment, the number of cells with impaired chromatin segregation and/or micronuclei was reduced, albeit not significantly, but misaligned chromosomes were significantly reduced (*p* < 0.005, Two-Way ANOVA). As expected, treatment with the SAC inhibitor NMS-P715 reduced the mitotic timing with respect to untreated samples (*p* < 0.001; Kruskal–Wallis with Dunn´s post-test) (Supplementary Figure S4d). However, contrary to 32D cells, HEL cells treated with ruxolitinib did not increase the mitotic timing (*p* > 0.05; Kruskal–Wallis test with Dunn´s post-test) (Supplementary Figure S4d).

Next, we treated HEL cells for 4 h with ruxolitinib before challenging them with nocodazole to determine whether JAK2 inhibition could extend the mitotic arrest. The mitotic arrest in HEL cells treated with ruxolitinib did not differ from DMSO controls (p > 0.05; two-tailed t-test) (Supplementary Figure S4 e, f). Together, these data suggest that the mitotic impact of JAK2V617F expression in human hematopoietic cells may depend on the lineage, additional mutations, and/or the need for other trophic factors and thus vary in magnitude and mechanism.

### JAK2V617F-mutant CD34 + patient-derived cells showed reduced expression of master mitotic regulators Aurora kinase B, PLK1 and PP2A catalytic subunit

**Figure 7:**
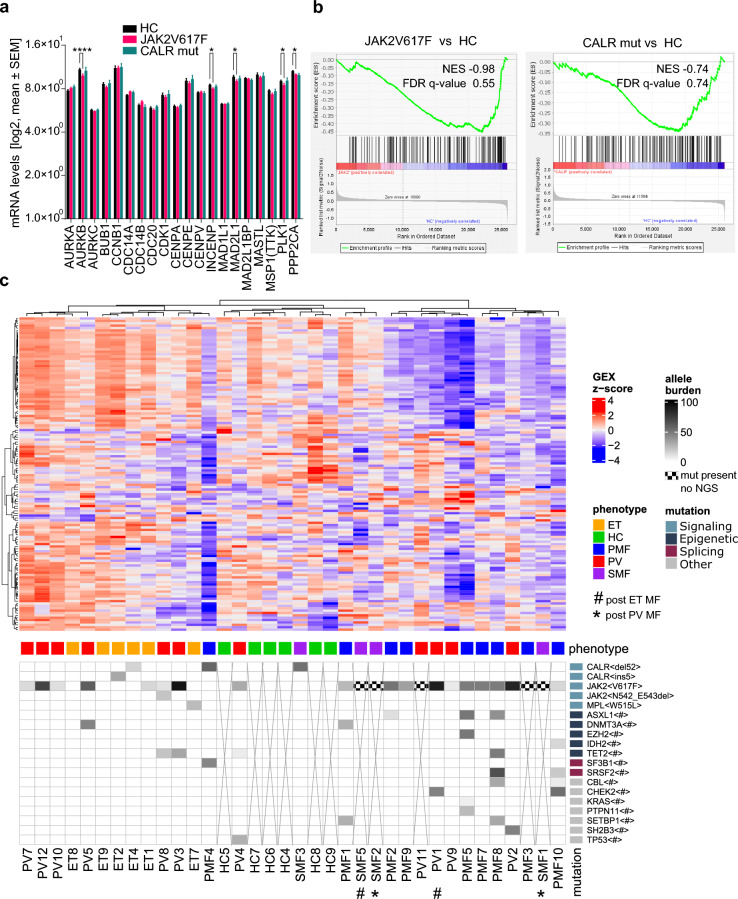
Differential expression of mitotic regulators in CD34^+^ enriched mononuclear cells from healthy controls (HC) and ET, PV, PMF, and SMF patients. (**a**) Gene expression profiles of SAC factors and key mitotic regulators were investigated using a previously published dataset^65^ in CD34^+^ enriched mononuclear cells obtained from healthy controls (HC) and Ph-neg. MPN patients carrying JAK2V617F or CALR mutations. Two-way ANOVA (Fisher´s LSD post-test;*p < 0.05; ****p < 0.001). (**b**) Gene set enrichment analysis of mitosis-related genes (see Supplementary Table 1) in JAK2V617 or CALRmut versus HC. (**c**) Unsupervised clustering of the expression profile of 132 mitotic genes (see Supplementary Table 1) in each Ph-neg. MPN subtype and HC samples. Allele burden of MPN-associated driver and bystander mutations are indicated by shades of gray. The processes affected by the mutations are indicated by a color code: signaling (light blue), epigenetic (dark blue), splicing (garnet), and others (grey). Samples lacking genotype by NGS are marked with x. Instances of JAK2V617F detection through alternative methods are indicated by hatching. The disease phenotype is indicated by a color code: ET (orange), healthy controls (HC; green), PMF (Blue), PV (red) and SMF (purple). Patients with secondary myelofibrosis post ET are indicated with (#) and post PV are indicated with (*). For more details, see Supplementary Figure S5.

CALRdel52 and JAK2V617F mutations deregulate gene expression in pathways related to inflammatory responses, proliferation, and differentiation in patient peripheral blood cells^63–65^. To investigate the impact of CALRdel52 and JAK2V617F mutations in the mitotic regulation of human hematopoietic stem and progenitor cells (HSPCs), we analyzed the transcriptional profile of important mitotic factors in HSPCs derived from ET, PV, PMF, and SMF patients as well as healthy donors in a publicly available dataset generated by our group ^65^. Expression analysis unveiled that in JAK2 mutant CD34 + cells the master mitotic regulators Aurora kinase B, PLK1, and the catalytic subunits of PP2A were downregulated (*p* < 0.05, one-way ANOVA) (Fig. 7a). While the expression of various SAC factors remained unchanged, MAD2L1 (also called MAD2) and INCENP stood out as significantly downregulated in JAK2 mutant CD34 + cells (*p* < 0.05, one-way ANOVA) (Fig. 7a).

To assess a potential over-representation of mitotic-related genes (see Supplementary Table 1) between JAK2V617 or CALRmut versus healthy donors, we conducted a gene set enrichment analysis (GSEA) (Fig. 7b). Notably, JAK2 or CALR mutations did not affect the entire mitotic pathway, but rather selectively influenced the expression of specific key mitotic regulators.

Subsequently, we conducted an unsupervised hierarchical clustering analysis to assess whether the expression profiles of genes related to mitotic regulation (see Supplementary Table 1) revealed distinct hierarchical relationships between the disease groups (Fig. 7c, Supplementary Figure S5). The resulting heatmap exhibits a considerable degree of heterogeneity among HCs, which nevertheless grouped together and included two patients (SMF3 and PV4). In eight of the nine PMF patients, a subgroup of mitotic regulators including several SAC factors was downregulated (upper part of the heatmap Fig. 7c, Supplementary Figure S5, Supplementary Table 3, Supplementary Figure S9). These PMF patients clustered closely with four PV and one SMF patients, all of whom exhibited analogous downregulation of this subgroup of mitotic regulators (Supplementary Table 3). These thirteen patients have a heterogeneous mutational profile with most of them carrying JAK2 mutations and displaying a moderate to high allele burden and a higher abundance of bystander mutations. Only one patient carried a CALR mutation. All ET patients clustered together intermingled with six out of eleven PV patients and showed enhanced expression of the above subgroup of mitotic regulators, including SAC factors (upper part of the heatmap Fig. 7c, Supplementary Figure S5, Supplementary Table 3, Supplementary Figure S9). These twelve patients showed a high heterogeneity regarding driver gene type and allele burden, also encompassing triple-negative patients. These data suggest that alterations in mitotic regulation could contribute to the pathology and phenotypic definition of MPN diseases.

## Discussion

The identification of (driver) mutations in JAK2, CALR, and MPL as well as bystander mutations, together with the characterization of crucial CALR and JAK2 canonical and non-canonical pathways, has provided substantial insight into the pathogenesis of Ph-neg. MPNs. However, it is still unclear how these malignancies evolve into more aggressive diseases^5^, such as secondary MF, secondary AML^7^, and secondary solid tumors^8,66^. Although karyotype aberrations compatible with mitotic failure are found at diagnosis^4,17,18^, accumulate at blast-phase transformation^19–22^, and are linked with decreased survival^23–25^, the process of mitotic chromatin segregation in this group of pathologies has not been studied in as much detail as in other diseases. We hypothesize that, as in solid cancers, mitotic errors as chromosome bridges, lagging chromosomes, and micronuclei, induced by driver Ph-neg. MPN mutations contribute to the disease progression and phase transition from chronic to acute MPN states. Our data confirm that CALRdel52 and JAK2V617F mutants disrupt the molecular regulation of mitosis, leading to errors in chromatin segregation. Furthermore, we find that CD34 + Ph-neg. MPN patient-derived cells show altered expression of important mitotic regulators and SAC factors. These data suggest that mitotic abnormalities contribute to disease progression and phase transition from chronic to acute MPN states.

We demonstrate that murine 32D^MPL^ cells transduced with CALRdel52 or JAK2V617F show stress-sensitive and error-prone mitosis, resulting in increased numbers of chromatin bridges, lagging chromosomes, and micronuclei. These mitotic errors are typically sources of aneuploidy and CIN and are linked to the acquisition and evolution of heterogeneous karyotypes^31,32^, as well as cGAS-STING-driven inflammation^67,68^ as a mechanism of malignant transformation. Strikingly, even mild karyotype abnormalities are involved in carcinogenic processes by conferring the aggressive malignant clones the genomic flexibility to survive and proliferate^31^. Lagging chromosomes, regardless of whether complete chromosomes or fragments can form micronuclei susceptible to massive genomic rearrangements by chromothripsis^69^, which in turn promotes genome changes favoring carcinogenesis^32,70^. Additionally, micronuclei resulting from missegregation events have been linked to epigenetic alterations in gene promoters critical for cell survival, differentiation, and proliferation^33^, alterations which indeed are present in a wide range of hematopoietic malignancies, including Ph-neg. MPNs^71,72^. Importantly, our data indicate that JAK2 inhibition with ruxolitinib at least partially protects 32D^MPL^ cells against the mitotic defects produced by SAC malfunction. This effect is stronger in CALRdel52 and JAK2V617F mutant cells. Furthermore, the mitotic defects in human JAK2V617F-expressing HEL cells induced after MPS1 inhibition are ameliorated under treatment with ruxolitinib, which however does not impact the SAC function of these cells. This suggests that the mitotic impact of CALRdel52 and JAK2V617F mutations may depend on the hematopoietic cell type, the presence of additional mutations (HEL cells harbor as well mutated p53), and/or the need for cytokines. It is also possible that HEL cells need a longer incubation time in ruxilitinib than 32D to obtain similar results. Altogether these results support the idea that CALRdel52 and JAK2V617F mutations are involved in mitotic control and identify the process of mitosis as a potential therapeutic target for Ph-neg. MPNs.

Our findings suggest that the stress-sensitive and error-prone mitosis in CALRdel52 or JAK2V617F murine cells is due to defects in the SAC. CALRdel52 and JAK2V617F expression induces defects in the SAC function in 32D^MPL^ cells reducing the time of mitotic arrest. There is no obvious correlation between the length of the mitotic arrest and the cell fate as reported for other cancer cells^47^. Moreover, our data from 32D^MPL^ (EV) cells treated with increased amounts of TPO support our hypothesis that mitotic differences are mainly driven by the analyzed oncogenes JAK2V617F and CALRdel52 and indicate that there might be only a partial contribution from MPL receptor-dependent signaling. Our experiments also show that the SAC is defective in human TF-1 cells expressing CALRdel52 or JAK2V617F. The SAC factors BubR1 or Bub1 are frequently downregulated in AML^11,12,51^. The expression of the BCR::ABL oncogene, a hallmark of CML, is accompanied by reduced Bub1, Bub3, BubR1, and Mad2 expression resulting in mitotic checkpoint defects that lead to CIN in murine 32D cells^15^. In contrast to this, our findings indicate that protein expression of critical SAC factors is unchanged in CALRdel52 and JAK2V617F transduced murine cells. Instead, the recruitment of several SAC factors to non-attached kinetochores is compromised or unbalanced. Here, it is important to note that mutant CALR or JAK2 overexpression induces recruitment defects for many SAC regulatory factors. These abnormal protein recruitments have similar trends for some factors such as MPS1 and BUBR1, but opposite trends for other factors such as CDC20, cyclin B, or MAD1. This indicates that the mitotic errors produced are similar, but the molecular mechanisms that mediate them might be different and complex in nature.

Subcellular localization and activity of critical SAC factors are often controlled by their phosphorylation status, which largely depends on the crosstalk between Aurora kinase B, PLK1, MPS1, and the multimeric phosphatase PP2A^26,28^. Yet, our analysis does not detect protein expression changes in the levels of these key regulators in murine CALRdel52 and JAK2V617F 32D cells. Therefore, it is conceivable that SAC defects in the presence of CALRdel52 or JAK2V617F are mediated by a change in the activation status of these or other mitotic regulators such as CDK1, WEE1, and PP1, which will be investigated in the future.

In humans, abnormal expression of mitotic regulators has been linked to disease initiation, progression, pathology, and prognosis of AML, CML, and MDS^16^. For example, Aurora kinases and PLK1 are upregulated in human leukemia cell lines and patient samples with AML and other myelodysplastic syndromes^13,73–76^. Accordingly, we find that PLK1, together with Aurora kinase B and the catalytic subunit of PP2A, are downregulated in JAK2 mutant CD34 + patient-derived cells. In these samples, unsupervised hierarchical clustering using the expression profiles of a large group of mitotic regulators, including most SAC factors, shows a decent separation of patients according to their phenotype with respect to healthy controls. A subgroup of these mitotic genes exhibited significant downregulation in a large proportion of the MF (“late” disease) patients, but not the ET and PV (“early” disease) patients, which in turn, showed enhanced expression of this subset of genes (Supplementary Table 3). Most of these genes are involved in several cellular processes, but all have been associated to some extent with mitotic activities (Supplementary Figure S9). They act at several time points during mitosis from shortly before the onset of prophase until the end of cytokinesis. Importantly, among them, we find master mitotic kinases (Aurora kinase A and B, PLK1, CDK1, NEK2, MASTL), important centromere/kinetochore components (CENPA, CENPP, CENPU, CENPQ, CENPK, ITGB3BP, NUF2, NDC80, Nup107), key spindle components (TPX2, KIF18A, KIF2C, SPC25, RANGAP1), and crucial SAC factors (BUB1, MPS1(TTK1), Cyclin B, INCENP, MAD2L1, CDC20). Collectively, these findings strongly indicate that a part of the mitotic regulatory network, including the SAC, is altered in Ph-neg. MPNs.

Importantly, the estimated leukemic transformation rate is 1–4% for ET, 3–7% for PV, and 9–13% for PMF^77^ and thus not negligible, although most MPN patients do not progress to leukemia. Our data suggest that both CALR mutant and JAK2V617F induce error-prone mitosis and that the expression of important mitotic regulators at the chronic stage of MPN is abnormal. Research to find the genetic or environmental factors that protect against or lead to phase transition will undoubtedly be relevant for the treatment of these diseases.

Elucidating the mechanisms through which expression changes of these factors contribute to the distinct phenotypic variances observed across these pathologies remains a complex challenge. Intriguingly, the analysis of the expression profile of mitotic regulators also groups two Ph-neg. MPN patients with no detected mutations in the most prominent MPN-associated genes according to the disease phenotype. Thus, mitotic regulators may also have prognostic and therapeutic significance in the Ph-neg. MPN context. Alternatively, this might indicate that mitotic imbalances are the necessary background to facilitate the emergence of Ph-neg. MPN.

In summary, our data suggest that CALRdel52 and JAK2V617F mutations induce chromatin segregation errors by disturbing the molecular control of mitosis in murine myeloid cells while CD34 + Ph-neg. MPN patient cells display a perturbed expression profile of a subset of important mitotic regulators, including BUB1, MAD2L1, INCENP, CDC20, CDK1, PLK1, and Aurora A/B. Recent evidence suggests that early JAK2V617F driver mutation acquisition in the first decade of life, accompanied by later genomic events, paves the way toward MPN in adulthood^78^. In this scenario, it is conceivable that small increases in the incidence of chromatin segregation defects, as observed here upon expression of JAK2 or CALR mutations, could have, after numerous mitotic divisions, a disruptive potential even in the absence of chemical stress. This could be aggravated by the countless chemical insults we are exposed to in daily life promoting cytogenetic abnormalities and additional inflammation via the cGAS-STING pathway that could favor disease progression and phase transition from chronic to acute MPN states.

## Methods

### Cell lines

Murine 32D cells (DSMZ, Braunschweig, Germany) were cultured in RPMI-1640 medium (PAN Biotec, Aidenbach, Germany) supplemented with 10% fetal calf serum (FCS) and 1% Penicillin–Streptomycin and 10% WEHI culture supernatant at 37 °C with 5% CO2. Human TF-1 cells (DSMZ, Braunschweig, Germany) were cultured in RPMI-1640 medium supplemented with 20% FCS, 1% Penicillin, and 2 ng/ml GM-CSF (Immunotools, Friesoythe, Germany). No additional thrombopoietin was added to either cell line. To generate 32D MPL-HA (32D^MPL^) and TF-1 MPL-HA (TF-1^MPL^) cells, 32D or TF-1 cells were transduced with the pMSCV-MPL-HA-IRES-puromycin vector followed by a second transduction with the empty pMSCV-IRES-GFP vector or pMSCV-IRES-GFP containing the JAK2V617F or the CALRdel52 oncogene as described before^79^. The transduction of the TF-1 cells was performed as follows: First, human TF-1 cells were retrovirally transduced with the ecotropic Scl7a1 (Eco) receptor, which made them susceptible to infection with murine retroviruses, and cells were positively selected with neomycin. Retroviruses, which carried the pMSCV-MPL-HA vector, were produced using PlatE cells as previously described^35^. Next, the TF1 Eco cells were transduced with a pMSCV-MPL-HA-generated (TF-1^MPL^) retrovirus and positively selected with puromycin. Finally, the cells were transduced with retrovirus carrying either the JAK2V617F or CALRdel52 mutation^80^.

### Live-cell imaging

To avoid Z-focal plane drop-out in solid media, non-adherent hematopoietic 32D, TF-1, or HEL cells were seeded in liquid medium in 8- or 18-well chambers (Ibidi, Gräfelfing, Germany), allowed to sediment for 30 min, and imaged in multi-position mode every 1.5–2 min for more than 15 h. Recording was performed with a 20 × 0.75NA objective using the fluorescence-widefield mode of an X-light spinning disk (CrestOptics, Roma, Italy) mounted on a Nikon Ti2 Eclipse microscope (Nikon, Melville, NY, U.S.A.) with the environmental control system UNO-T-H-CO2 (Okölab, Ottaviano, NA, Italy). Samples were illuminated with a LED light engine SpectraX (Lumecor, Beaverton, OR, USA) at very low intensities (< 3% of line source power and < 1 s acquisition per channel), through mCherry filter sets for chromatin imaging (mCherry), and GFP for assessing single cell expression of the EV-, CALRdel52- or JAK2V617F.

### Quantitative analysis of mitotic timing and mitotic errors

Cells seeded in 8- or 18-well chambers (Ibidi, Gräfelfing, Germany) were treated with 200 nM of doxorubicin^36^ (Calbiochem, St. Louis, MO, USA) or 3 µM of the MPS1-inhibitor NMS-P715^37^ (Merck KGaA, Darmstadt, Deutschland) 1 h before starting multi-position long-term (20 h) live-cell imaging as indicated above. Dwell time analysis of early mitosis (prophase to anaphase onset) was performed using CecogAnalyzer 1.5.2 (https://cellcognition-project.org/software.html)^81^ as described before in^82^. Due to the high in mobility of the cells, the available algorithms for automatic tracking typically select many erroneous trajectories as valid mitotic events. A code in R-Studio (provided by Dr. Ramona Jühlen, Institute of Biochemistry and Molecular Cell Biology, Faculty of Medicine, RWTH Aachen University, Aachen, Germany) was used to obtain early mitosis dwell times by unbiasedly discarding erroneous trajectories. This code filters the CellCognition numerical output tables for trajectories whose parameters are unambiguously mitotic. The frequency and type of chromatin segregation errors were then evaluated by visual scoring and classification on image galleries of valid mitotic trajectories.

To test the effect of JAK2 inhibition in the mitotic phenotypes, cells seeded in 8-well chambers (Ibidi) were treated with DMSO or 1 µM of ruxolitinib (dissolved in DMSO, Selleckchem, Houston, TX) for 4 h and 3 µM of the MPS1-inhibitor NMS-P715 for 1 h before starting multi-position long-term live-cell imaging as indicated above. The analysis of dwell times in mitosis and the frequency and type of chromatin segregation errors in 32D cells was performed as indicated above. To test the effect of JAK2 inhibition on the mitosis of human acute erythroid leukemia HEL cells. The cells were seeded in 8-well chambers (Ibidi) containing growth medium with 200 nM SiR-Hoechst (SiR-DNA; Spirochrome) to label the chromatin. Cells were treated with DMSO or 1 µM of ruxolitinib for 4 h and with DMSO or 3 µM of the MPS1-inhibitor NMS-P715 for 1 h before starting multi-position long-term live-cell imaging as described above. Expert biologists extracted and annotated mitotic trajectories using Open FiJi (ImageJ 1.53c/ Java 1.8.0_172) to determine dwell times in mitosis and the frequency and type of chromatin segregation errors.

### Spindle assembly checkpoint challenge

Cells were seeded into 8- or 18-well chambers (Ibidi, Gräfelfing, Germany) and treated with 100 ng/ml (330 nM) nocodazole immediately before starting multi-position long-term live-cell imaging. To test the effect of JAK2 inhibition on the SAC, cells were treated with DMSO or 1 µM of ruxolitinib (dissolved in DMSO, Selleckchem, Houston, TX) for 4 h before starting multi-position long-term live-cell imaging. To test the effect of TPO on the SAC, 32D^MPL^ (EV) cells were treated with 0, 10, 50, or 100 ng/ml of TPO (Miltenyi Biotec, Bergisch Gladbach, Germany) for 4 h before starting multi-position long-term live-cell imaging. To analyze the duration and fate of the mitotic arrest, the records were visually inspected by an expert biologist using Open FiJi (ImageJ 1.53c/ Java 1.8.0_172)^83^, annotating time from chromatin condensation at prophase entry to mitotic slippage/chromatin segregation. Cells remaining in mitotic arrest until the end of the record were not taken into account.

### Immunofluorescence

32D^MPL^ cells were centrifuged onto coverslips (1000 rpm), and fixed for 10 min with 4% PFA in PBS. After quenching for 5 min in 50 mM NH_4_Cl in PBS-Tween, samples were washed in PBS-Tween supplemented with 1 mM MgCl_2_ and 0.1 mM CaCl_2_ and blocked in 5% BSA in PBS with MgCl_2_ and CaCl_2_ for 30 min. Samples were incubated with the primary and secondary antibodies for 2 h with three washings of 5 min in between (see Supplementary Table 2) and mounted with Mowiol. The imaging was carried out on a Nikon Ti2 Eclipse microscope (see above) using a Lambda Oil 60 × NA 1.4 objective from Nikon with Z-stacks spanning the height of the cell monolayer from random sample fields. The MAD2xl antibody was generated as follows: *Xenopus laevis* MAD2 was cloned into a pET28a vector, expressed in BL21 (DE3) cells, purified with Ni–NTA-Agarose, and used for antibody production in rabbits.

For quantitative immunofluorescence, fixation and acquisition were done as described above with the cells treated with 200 ng/ml of nocodazole for 3 h prior to centrifugation. The analysis was done with a custom pipeline, where Open FiJi converted Nikon “.nd” image files to TIFF files, on which Cellprofiler^84^ performed segmentation followed by signal quantitation. Shortly, the Open FiJi Batch Macro tool generated SUM projections of random sample fields acquired and converted them to multi-channel TIFF files. In these files, mitotic arrested cells were visually identified and extracted as single-cell multi-channel TIFFs. Then, a custom CellProfiler analysis pipeline (see Supplementary materials) segmented the chromatin region (identified by the mCherry signal) and the kinetochore region (CREST staining) and quantified the bulk fluorescence intensities of the different antigens. From these, the relative mean kinetochore intensity was calculated as the ratio of the intensity for each antigen to the CREST signal^30,53,61^. Calculations, plotting, and statistical analysis were done in Excel (Microsoft Corporation, Redmond, USA), and GraphPad Prism software version 9.0 (GraphPad Inc., La Jolla, USA).

### Western blot

Western blots of whole cell lysates were performed in Lämmli buffer at 25 mA, using TRIS–glycine buffer for transfer 3 h at 100 mA at 4 °C. The membranes were stained with Ponceau S for quality control and marker labeling, blocked in 5% milk powder in PBS-Tween and after primary (O/N at 4 °C) and secondary antibody (1 h) incubation and three times washing in between. The blots were developed using the ECL substrate Western Bright Quantum (Advansta) on an ImageQuant LAS-4000 system (Fuji). The analysis was done in Open FiJi with the gel analysis tool. Shortly, the signal was measured as the area under the profile of intensity of each band over the surrounding background. Then, the signal ratio of each band with respect to its corresponding loading control (Actin or GADPH) was calculated. Calculations, plotting, and statistical analysis were done in Excel and GraphPad Prism software 9.0.

### mRNA chip seq and bioinformatics

A previously published dataset (GSE174060) using CD34-enriched MPN patient-derived and healthy control (HC)-derived PBMCs and bone marrow mononuclear cells has been utilized for gene expression analysis and gene set enrichment analysis (GSEA)^65^. For GSEA, PBMCs carrying JAK2V617F and CALR mutations derived from patients diagnosed with ET, PMF, and SMF, as well as healthy control cells have been included in the analysis with the GSEA software of UC San Diego and Broad Institute^85,86^. The gene set is given in Supplemental Table 1. Unsupervised clustering of mitotic genes was performed using all patients and HCs. Unsupervised clustering on the z-score transformed gene expression values of the mitotic gene set for all patients and HCs was performed. The clustering distance was set as the Pearson correlation distance while “ward.D2” (set as the method parameter in the hclust function in R) was the clustering method used. We should note that, before calculating the z-scores, expression values were averaged out for genes that appeared more than once in the data.

Based on the heatmap presented in Fig. 7c, the dendrogram was limited to the genes corresponding to the "upper branch". These are the genes that are generally downregulated in the PMF/SMF/PV1/11/9 cohort. An enrichment analysis of these 47 genes was performed using the R programming language, with the gprofiler2 package ^87^. Default parameters were used, except for the p-value correction methods where the false-discovery rate was used as the correction method. Only terms with adjusted *p*-values below a threshold of 0.05 are shown and plotted.

### Statistical analysis

For all experiments a p-value under 0.05 was considered to be statistically significant. No statistical methods have been used to predetermine sample sizes. All the data are derived from at least three independent experiments. The data were tested for normality with the D’Agostino & Pearson test or Shapiro–Wilk test (when *n* =  < 3) to decide whether a parametric or non-parametric test is necessary. One-way ANOVA or Two-Way ANOVA with Dunnet post-test or two-tailed t-test were applied to normal distributions. When normality could not be assumed, statistical significance was determined using a One-way ANOVA (Kruskal–Wallis test with Dunn´s posttest). For categorical response type data, the Fischer test was used^88^. Plots, Superplots, and statistical significance of data were performed using the GraphPad Prism software version 9.0 (GraphPad Inc., La Jolla, USA). Statistical significance for each condition can be found in the figures and/or figure legends.

### Supplementary Information

Supplementary Information 1.

Supplementary Information 2.

## Data Availability

The datasets used during the current study are available from the corresponding author upon reasonable request. 23. The dataset from the CD34-enriched MPN patient-derived and healthy control (HC)-derived PBMCs and bone marrow mononuclear cells [65] is publically available at the Gene Expression Omnibus (GSE174060) (https://www.ncbi.nlm.nih.gov/geo/query/acc.cgi?acc=GSE174060).
