# Supplementary material for: Calreticulin and JAK2V617F driver mutations induce distinct mitotic defects in myeloproliferative neoplasms
Source: Sci Rep. 2024 Feb 2;14:2810. doi: 10.1038/s41598-024-53240-8 (PMC10837458; doi:10.1038/s41598-024-53240-8)
Supplement: Supplementary file 1 — Supplementary Information 1. [file 41598_2024_53240_MOESM1_ESM.pdf]

Calreticulin and JAK2V617F driver mutations induce distinct mitotic defects in myeloproliferative neoplasms

Kristin Holl[1], Nicolas Chatain[2,3], Susanne Krapp[1], Julian Baumeister[2,3], Tiago Maié[4], Sarah Schmitz[1], Anja Scheufen[1], Nathalie Brock[1], Steffen Koschmieder[2,3] and Daniel Moreno-Andrés[1*]

1. Institute of Biochemistry and Molecular Cell Biology, Faculty of Medicine, RWTH Aachen University, Aachen, Germany.
2. Department of Hematology, Oncology, Hemostaseology, and Stem Cell Transplantation, Faculty of Medicine, RWTH Aachen University, Aachen, Germany
3. Center of Integrated Oncology Aachen Bonn Cologne Düsseldorf (CIO ABCD), Aachen, Germany.
4. Institute for Computational Genomics, Joint Research Center for Computational Biomedicine, Faculty of Medicine, RWTH Aachen University, Aachen, Germany.
5. Corresponding author(*): Daniel Moreno-Andrés (dmoreno@ukaachen.de)

Supplementary Figure S1

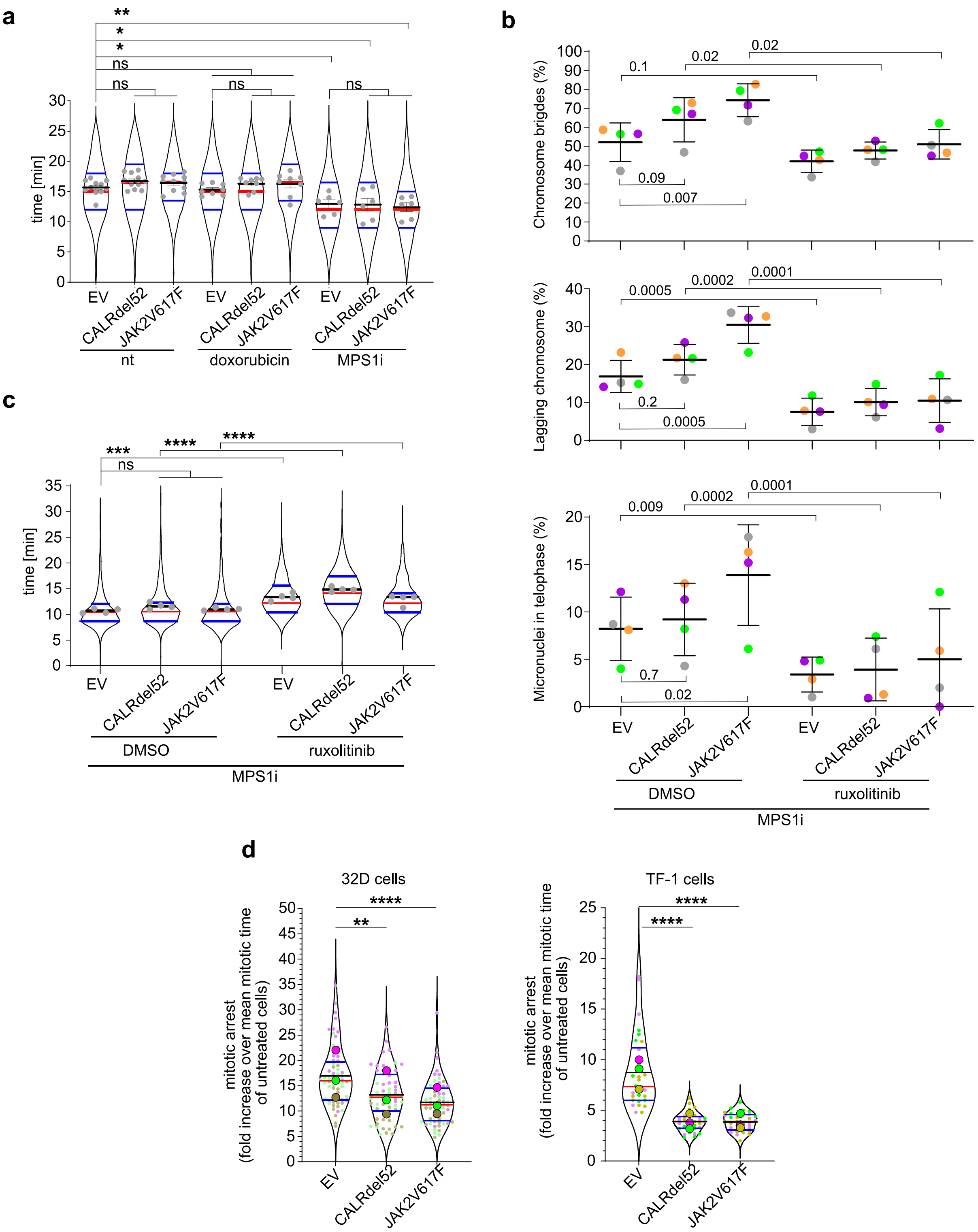

Supplementary Figure S1. CALRdel52 and JAK2V617F mutations do not affect mitotic duration in murine 32D^{MPL}. a) Superplots of the mitotic duration in control (EV), CALRdel52, and JAK2V617F transduced 32D^{MPL} cells expressing H2B-mCherry, untreated (nt) or treated with 200 nM doxorubicin or 3 µM MPSi. The violin superplots show pooled data from 6 to 11 independent experiments with 100 cells per experiment and condition. The thick grey dots indicate independent experiment means. Horizontal red lines indicate medians, black lines mean, and blue lines are quartiles of the pool. One-Way ANOVA *p<0.05 **p<0.01. b) Plots showing the percentage of chromatin bridges, lagging chromosomes and micronuclei in control (EV), CALRdel52 or JAK2V617F 32D cells in the presence of 3 µM MPS1 inhibitor NMS-P715 after treatment with DMSO or 1 µM ruxolitinib for 4 h (Four independent, color-coded experiments with >90 cells per experiment and condition, the horizontal black lines, and dispersion bars indicate means and SD of the means). The significance p-values were obtained with the exact Fisher test. c) Superplots of mitotic duration in control (EV), CALRdel52 or JAK2V617F 32D cells in presence of 3 µM MPS1 inhibitor NMS-P715 after treatment with DMSO or 1 µM ruxolitinib for 4h. The violin superplots show pooled data from 4 independent experiments with >90 cells per experiment and condition. The thick grey dots indicate independent experiment means. Horizontal red lines indicate medians, black line mean, and blue lines are quartiles of the pool. One-Way ANOVA *p<0.05 **p<0.01. d) Fold increase of mitotic time in arrest in 100 ng/ml nocodazole treated control (EV), CALRdel52 and JAK2V617F 32D^{MPL} or TF-1 cells over mean mitotic time of untreated cells. The violin superplots show three independent (color-coded) experiments with 20 cells per experiment and cell line. The thick colored dots indicate independent experiment means. Horizontal red lines indicate medians, black lines mean, and blue lines are quartiles of the pools. Kruskal-Wallis with Dunn´s post-test; **p<0.01 ****p<0.001.

Supplementary Figure S2

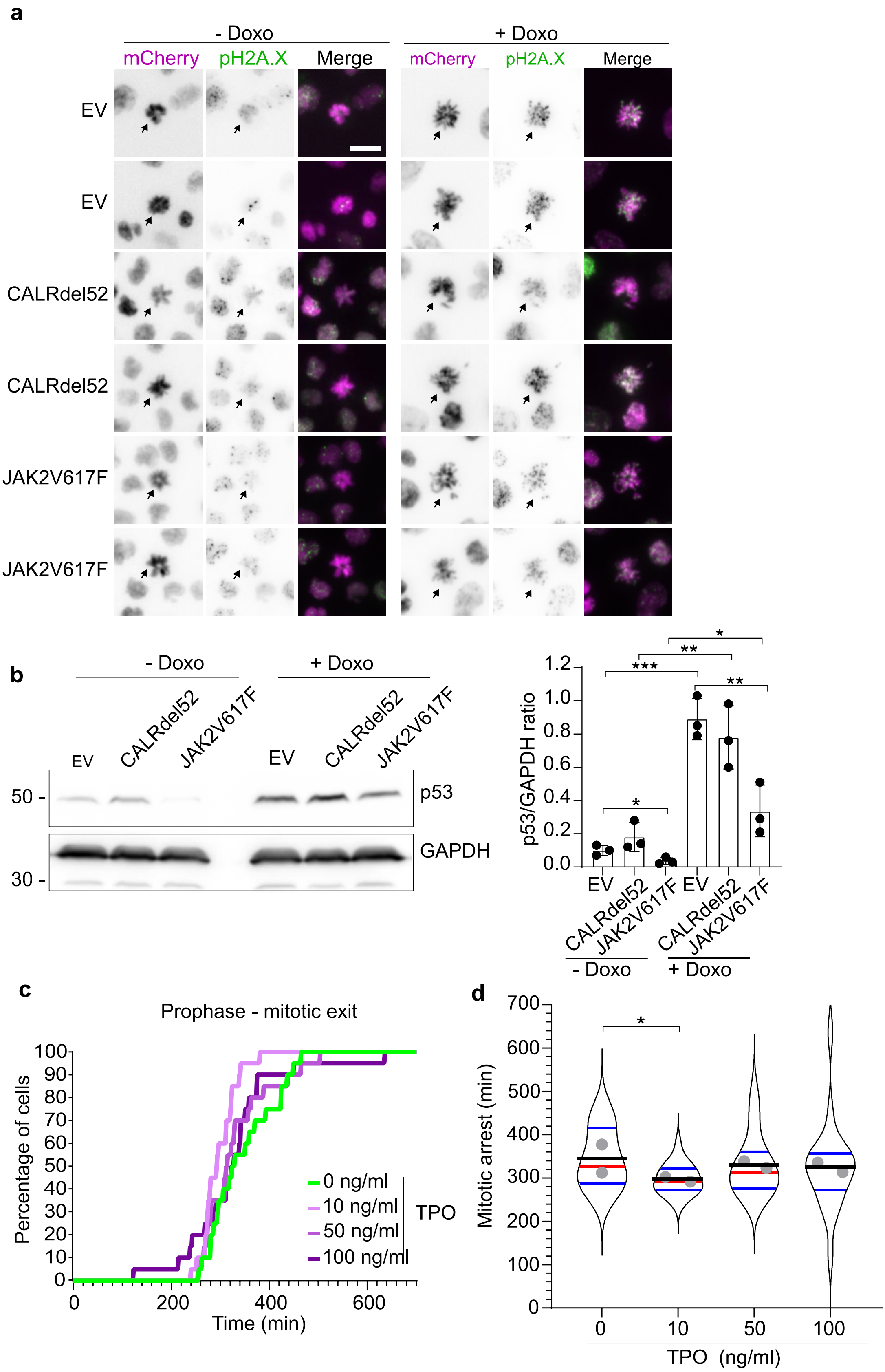

Supplementary Figure S2. **DNA damage and p53 levels are unchanged in mitotic control (EV), CALRdel52, or JAK2V617F transduced 32D^{MPL} cells.** a) Representative immunofluorescence images of untreated or doxorubicin-treated (30h, 200nM) prometaphase cells stained for DNA double-strand breaks with the γ-H2AX (H2AX S139ph). Scale bar 10 µm. b) Representative cropped western blots for p53 and GAPDH as loading control of whole cell extracts after being treated or not with doxorubicin (30h, 200nM). Quantitation of p53 protein expression normalized to GAPDH. The columns indicate the means, error bars SDs, and black dots individual data points of three independent experiments. Unpaired t-test; * p<0.05; ** p<0.01; ***p<0.01; ns p>0.05. For uncropped WB see Supplementary Figure S8. c) Plots show the fraction of control (EV) 32D^{MPL} cells cultured with the indicated concentrations of TPO for more than 4h that exit mitosis at the given time after mitotic entry in the presence of 100 ng/ml nocodazole. d) Superplots of mitotic duration in control (EV) 32D^{MPL} from (c). The violin superplots show pooled data from 2 independent experiments with 10 cells per experiment and condition. The thick grey dots indicate independent experiment means. Horizontal red lines indicate medians, black lines mean, and blue lines quartiles of the pool. Kruskal-Wallis test with uncorrected Dunn´s post-test; * p<0.05.

Supplementary Figure S3

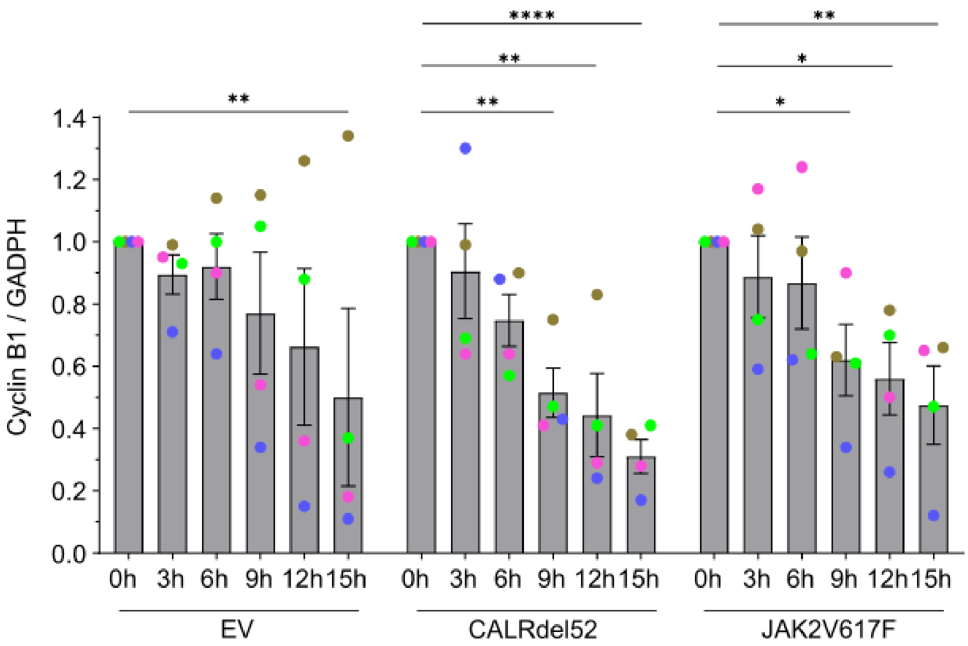

Supplementary Figure S3. Cyclin B1 degradation is faster in cells expressing CALRdel52 and JAK2V617F mutations. Cyclin B1 levels in cells, treated for 3 h with 200ng/ml nocodazole. Time points were taken after nocodazole wash out at t=0. Samples are, normalized to GAPDH and t=0. Columns indicate the means, error bars, SDs, and colored dots the values of four independent experiments. Two-way ANOVA with Dunnett post-test; * p<0.05; **p<0.01; ***p<0.001.

Supplementary Figure S4

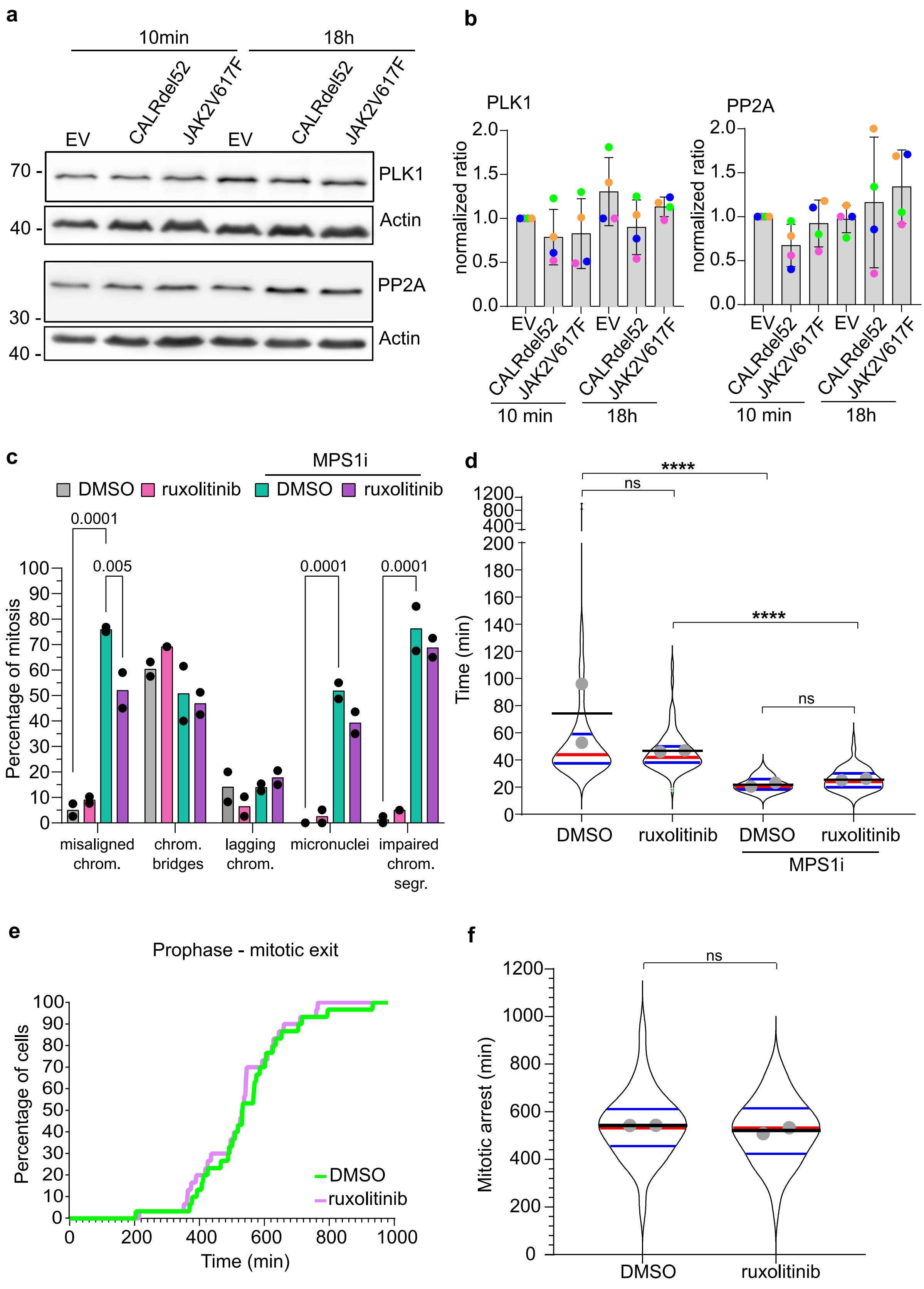

Supplementary Figure S4. CALRdel52 and JAK2V617F mutations do not affect protein expression of the mitotic regulators PLK1 and PP2A. a) Cropped western blots for PLK1, PP2A, and actin as loading control of whole cell extracts from cells at 10 min and 18 h after mitotic arrest induction with 200ng/ml nocodazole. b) Quantitation of the expression of PLK1 or PP2A normalized to actin and EV control. Grey columns indicate the means, the error bars the SDs, and the colored dots the values of four independent experiments. One-way ANOVA (Kruskal-Wallis test). For uncropped WB see Supplementary Fig. S8. c) Plots showing the percentage of chromatin bridges, lagging chromosomes and micronuclei in HEL cells in the presence of 3 µM MPS1 inhibitor NMS-P715 after treatment with DMSO or 1 µM ruxolitinib for 4 h (two independent experiments with >30 cells per condition, the bars indicate means and the dots the percentages of independent experiments). The significance p-values were obtained with Two-Way ANOVA. d) Superplots of mitotic duration in HEL cells from (c). The violin superplots show pooled data from 2 independent experiments with >30 cells per experiment and condition. The thick grey dots indicate independent experiment means. Horizontal red lines indicate medians, black lines mean, and blue lines are quartiles of the pool. Kruskal-Wallis with Dunn´s post-test; ns p>0.05 ****p<0.001. e) Fraction of HEL cells treated with DMSO or 1 µM ruxolitinib for 4 h that exit mitosis at the given time after mitotic entry in the presence of 100 ng/ml nocodazole. f) Mitotic duration in HEL cells from (c). The violin superplots show pooled data from 2 independent experiments with 15 cells per experiment and condition. The thick grey dots indicate independent experiment means. Horizontal red lines indicate medians, black lines mean, and blue lines are quartiles of the pool. Two-tailed t-test; * p<0.05.

Supplementary Figure S5

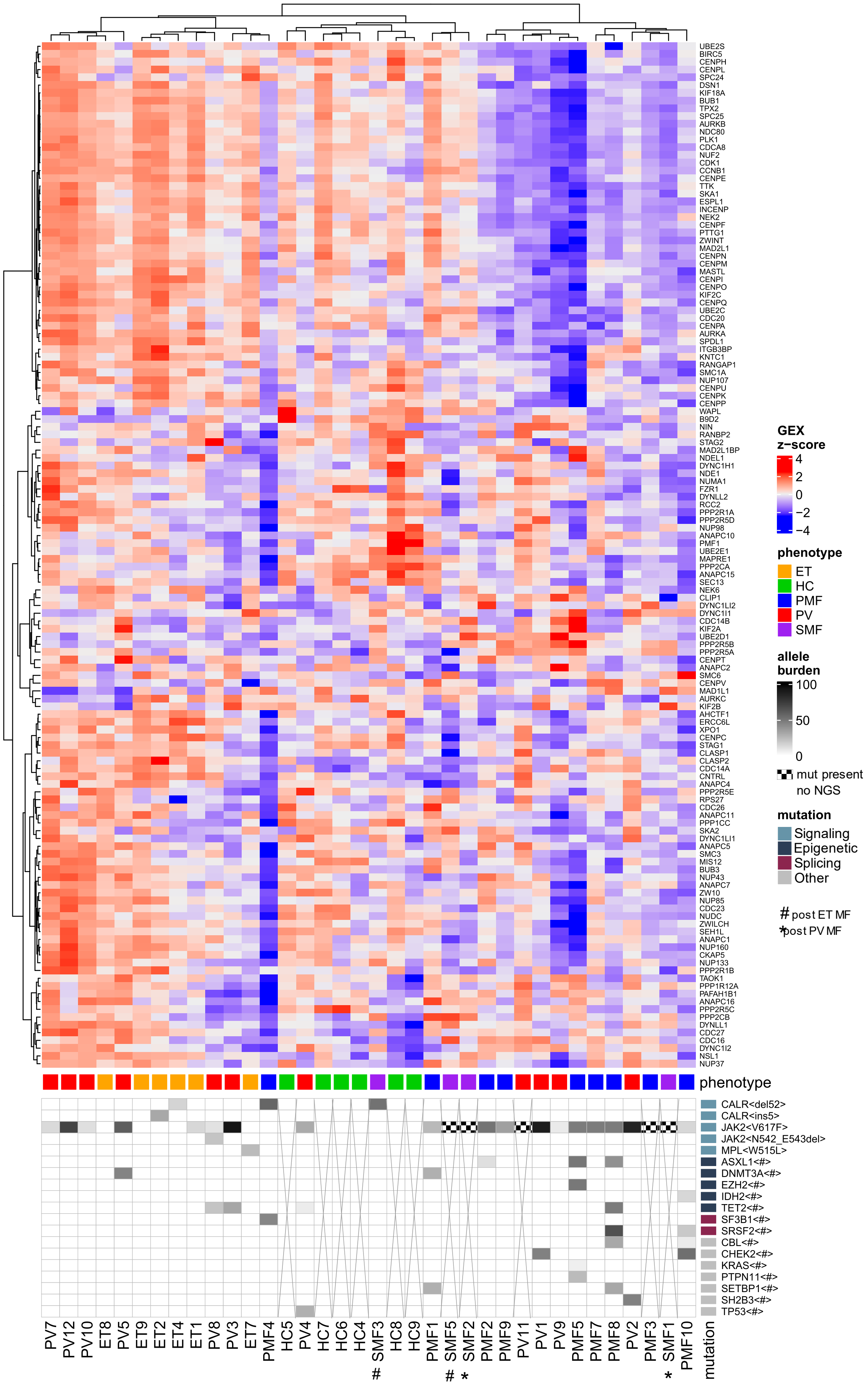

Supplementary Figure S5. Hierarchical clustering of differentially expressed mitotic genes in CD34+ enriched mononuclear cells separates healthy controls (HC) from ET, PV, PMF, and SMF patients. Heatmap of unsupervised clustering using the expression profile of 132 mitotic genes (see Supplementary Table 1) in each Ph-neg. MPN subtype and HC samples. Allele burden of MPN-associated driver and bystander mutations are indicated by shades of gray. The process affected by the mutations are indicated by a color code: signaling (light blue), epigenetic (dark blue), splicing (garnet), and others (grey). Samples lacking genotype by NGS are marked with x. Instances of JAK2V617F detection through alternative methods are indicated by hatching. The disease phenotype is indicated by a color code: ET (orange), healthy controls (HC; green), PMF (Blue), PV (red) and SMF (purple). Patients with secondary myelofibrosis post ET are indicated with (#) and post PV are indicated with (*).

Supplementary Figure S6

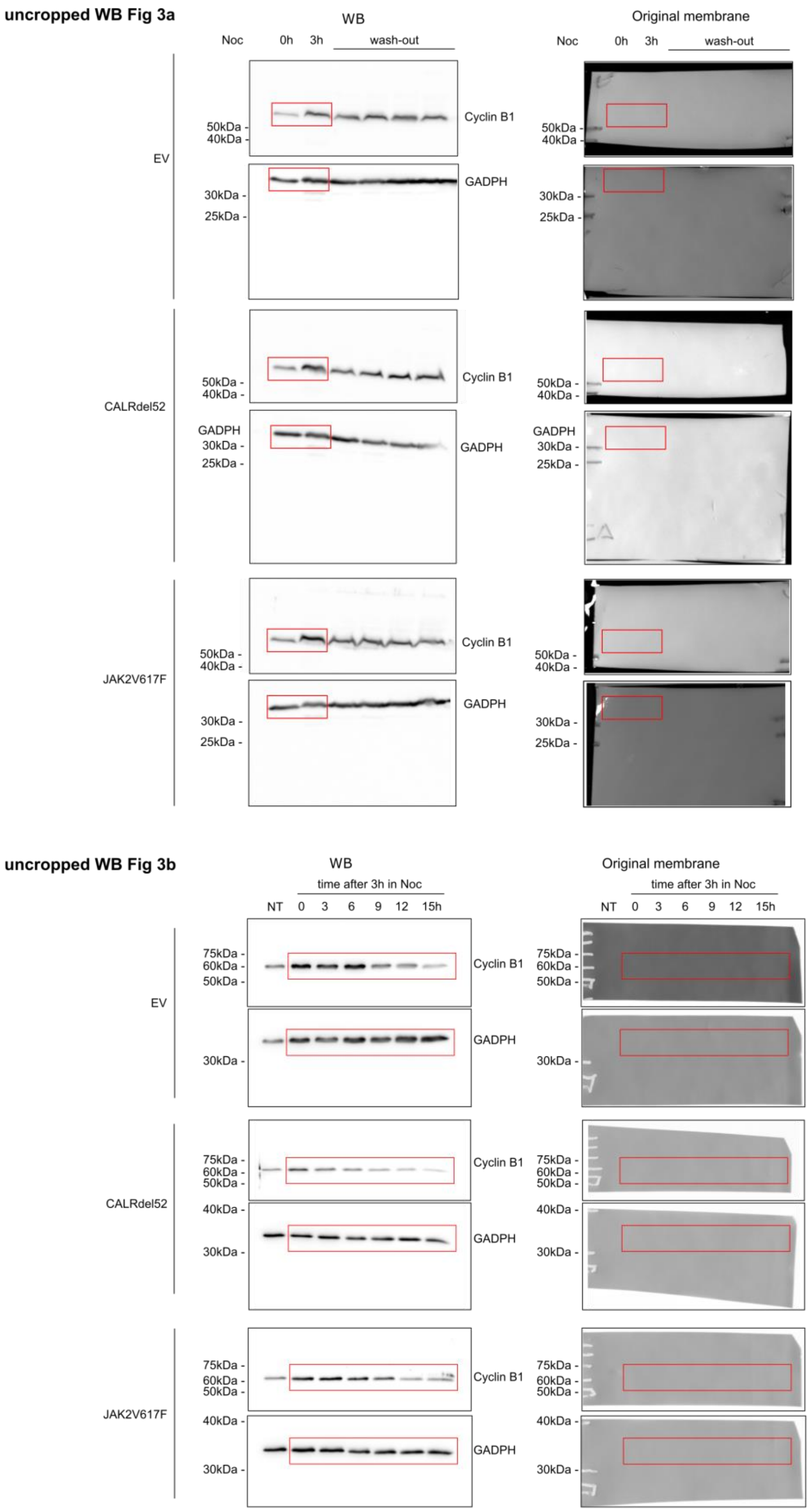

Supplementary Figure S6. Uncropped WB and original membranes from Figure 3a and b. The red rectangle indicates the cropped area displayed in the main and supplementary figures.

Supplementary Figure S7

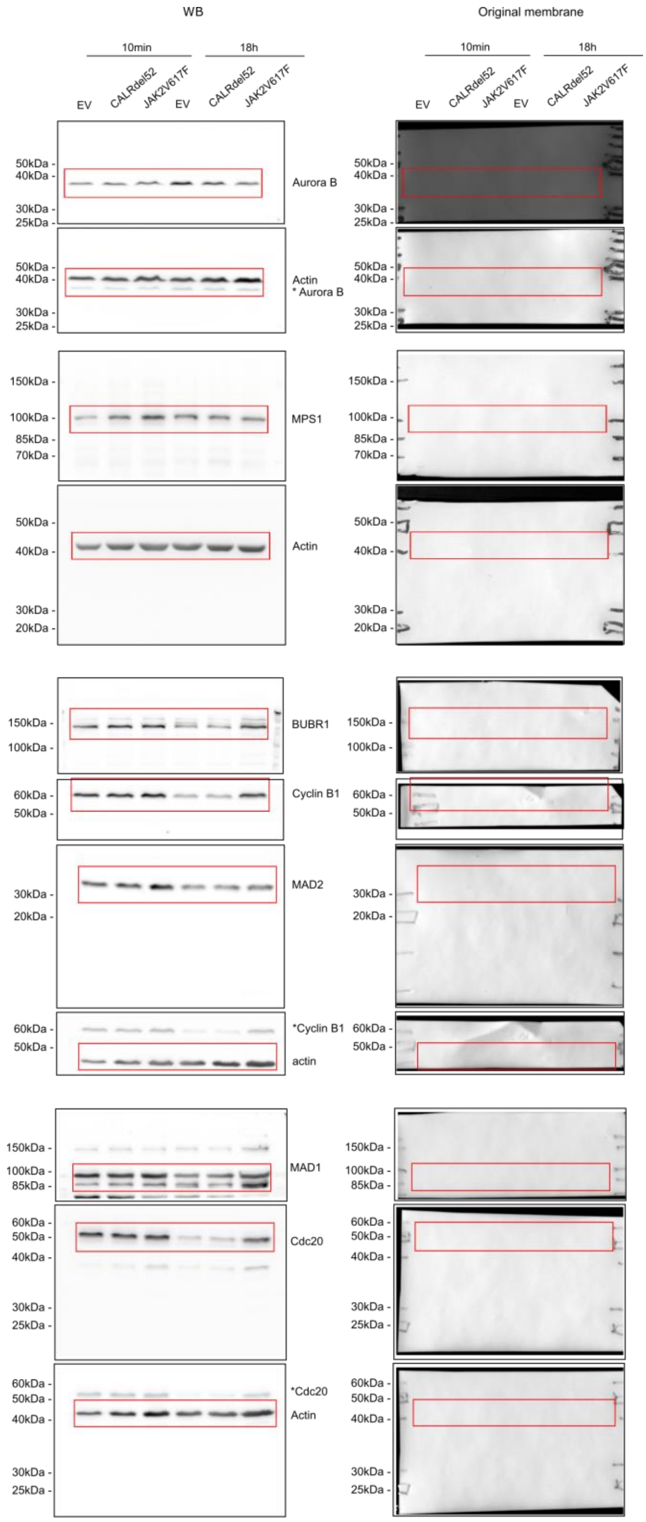

Supplementary Figure S7. Uncropped WB and original membranes from Figure 4a. The asterisks (*) indicate that the band at that height comes from the WB performed on the same membrane crop prior to the detection of the actin loading control. The red rectangle indicates the cropped area displayed in the main and supplementary figures.

Supplementary Figure S8

UNCROPPED WB Supplementary Figure S2b

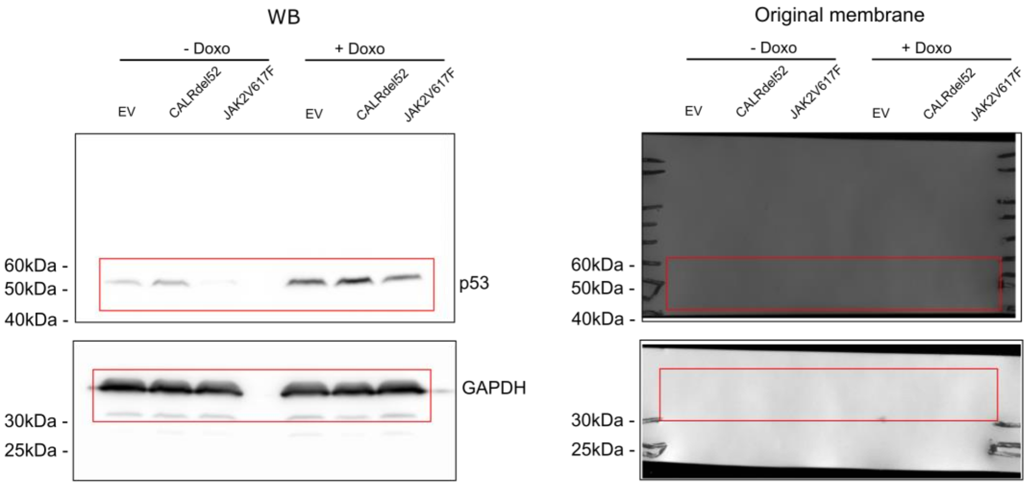

UNCROPPED WB Supplementary Figure S4a

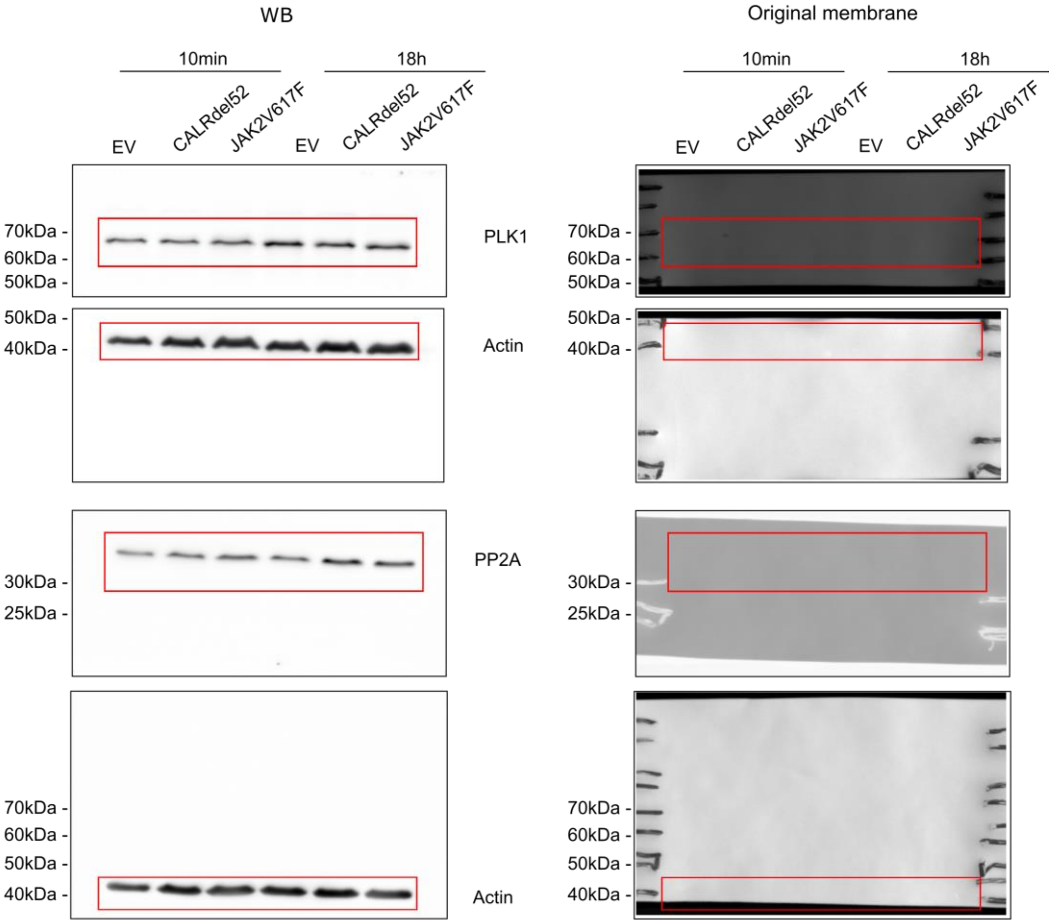

Supplementary Figure S8. Uncropped WB and original membranes from Supplementary Figure S2a and Supplementary Figure S4a. The red rectangle indicates the cropped area displayed in the main and supplementary figures.

Supplementary Figure S9

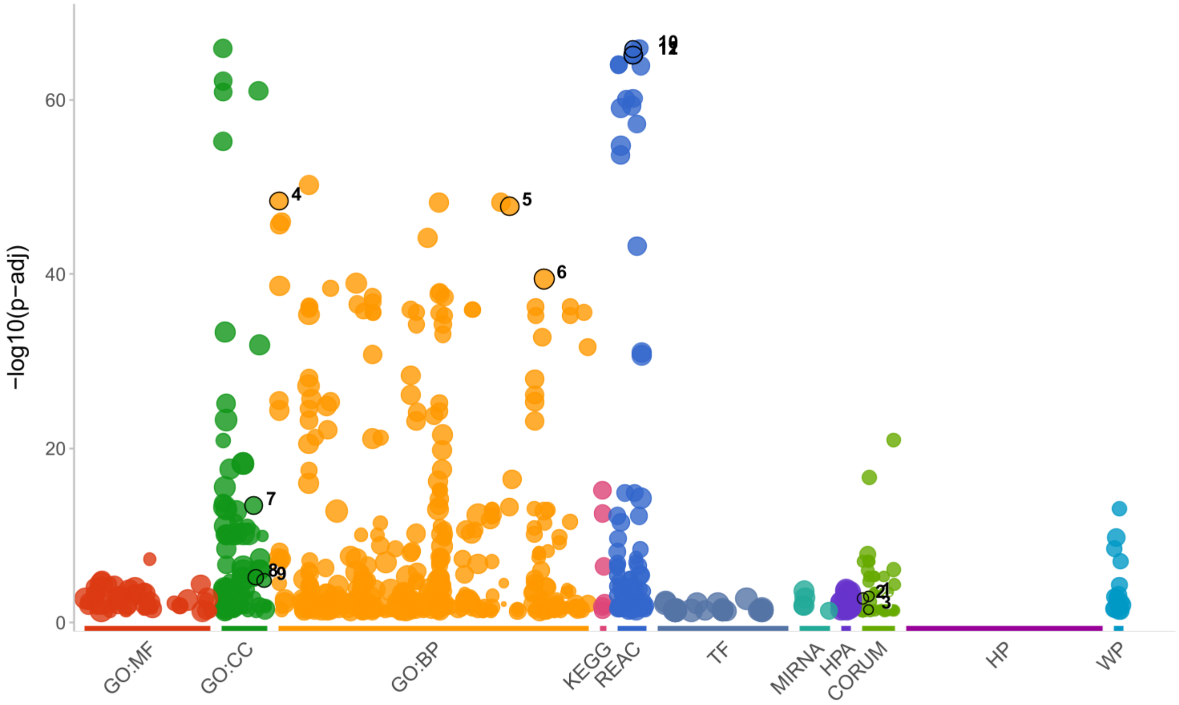

id	source	term_id	term_name	term_size		p_value
1	CORUM	CORUM:1116	CRM1−Survivin−AuroraB mitotic complex	3		9.4e−04
2	CORUM	CORUM:190	Mitotic checkpoint complex (MCC)	4		1.8e−03
3	CORUM	CORUM:1117	CRM1−Survivin mitotic complex	2		3.0e−02
4	GO:BP	GO:0000070	mitotic sister chromatid segregation	187		4.1e−49
5	GO:BP	GO:0140014	mitotic nuclear division	278		1.6e−48
6	GO:BP	GO:1903047	mitotic cell cycle process	757		3.6e−40
7	GO:CC	GO:0072686	mitotic spindle	184		3.8e−14
8	GO:CC	GO:0097431	mitotic spindle pole	38		6.2e−06
9	GO:CC	GO:1990023	mitotic spindle midzone	14		1.5e−05
10	REAC	REAC:R−HSA−69618	Mitotic Spindle Checkpoint	109		1.5e−66
11	REAC	REAC:R−HSA−2555396	Mitotic Metaphase and Anaphase	229		7.3e−66
12	REAC	REAC:R−HSA−68882	Mitotic Anaphase	228		7.3e−66

g:Profiler (biit.cs.ut.ee/gprofiler)

Supplementary Figure S9. Enrichment analysis for mitotic processes of the negative GEX z-score genes of PMF/SMF/PV1/11/9 cohort (Supplementary Figure S5 and Supplementary Table 3). Top: Manhattan-like-plot showing terms, color-coded by data source (see annotation below). The y-axis shows the adjusted p-values in the negative log10 scale. Each circle is a term, whereas the size of the circle denotes the term size. Bottom: Table highlighting the top 3 terms by adjusted p-value for their respective data source, related to the search term "Mitotic" or "mitotic". Annotation: GO - Gene Ontology, MF - Molecular Function, CC - Cellular Component, BP - Biological Process; REAC - Reactome, WP - WikiPathways; TF - TRANSFAC; MIRNA - miRTarBase; HPA - Human Protein Atlas; HP - Human phenotype ontology.

Supplementary table 1. Human Gene Set of important mitotic genes.
The table combines non redundant HALMARKS of the Molecular Signatures Database (MSigDB) for Mitotic spindle checkpoint (https://www.gsea-msigdb.org/gsea/msigdb/human/geneset/REACTOME_MITOTIC_SPINDLE_CHECKPOINT.html) and the Mitosis (https://www.gsea-msigdb.org/gsea/msigdb/cards/MITOSIS).

Human Gene Set Mitosis & SAC		
**NCBI (Entrez) Gene Id**	**Gene Symbol**	**Gene Description**
8379	MAD1L1	mitotic arrest deficient 1 like 1...
996	CDC27	cell division cycle 27 [Source:HG...
5048	PAFAH1B1	platelet activating factor acetyl...
23279	NUP160	nucleoporin 160 [Source:HGNC Symb...
55166	CENPQ	centromere protein Q [Source:HGNC...
54908	SPDL1	spindle apparatus coiled-coil pro...
29945	ANAPC4	anaphase promoting complex subuni...
5525	PPP2R5A	protein phosphatase 2 regulatory ...
3796	KIF2A	kinesin family member 2A [Source:...
5526	PPP2R5B	protein phosphatase 2 regulatory ...
55746	NUP133	nucleoporin 133 [Source:HGNC Symb...
7321	UBE2D1	ubiquitin conjugating enzyme E2 D...
54820	NDE1	nudE neurodevelopment protein 1 [...
23332	CLASP1	cytoplasmic linker associated pro...
79023	NUP37	nucleoporin 37 [Source:HGNC Symbo...
1781	DYNC1I2	dynein cytoplasmic 1 intermediate...
5527	PPP2R5C	protein phosphatase 2 regulatory ...
10403	NDC80	NDC80 kinetochore complex compone...
7514	XPO1	exportin 1 [Source:HGNC Symbol;Ac...
81929	SEH1L	SEH1 like nucleoporin [Source:HGN...
9183	ZW10	zw10 kinetochore protein [Source:...
8655	DYNLL1	dynein light chain LC8-type 1 [So...
51433	ANAPC5	anaphase promoting complex subuni...
332	BIRC5	baculoviral IAP repeat containing...
10726	NUDC	nuclear distribution C, dynein co...
8697	CDC23	cell division cycle 23 [Source:HG...
79019	CENPM	centromere protein M [Source:HGNC...
5905	RANGAP1	Ran GTPase activating protein 1 [...
22919	MAPRE1	microtubule associated protein RP...
2491	CENPI	centromere protein I [Source:HGNC...
80152	CENPT	centromere protein T [Source:HGNC...
5516	PPP2CB	protein phosphatase 2 catalytic s...
5518	PPP2R1A	protein phosphatase 2 scaffold su...
27338	UBE2S	ubiquitin conjugating enzyme E2 S...

25906	ANAPC15	anaphase promoting complex subuni...
4928	NUP98	nucleoporin 98 and 96 precursor [...
57122	NUP107	nucleoporin 107 [Source:HGNC Symb...
5528	PPP2R5D	protein phosphatase 2 regulatory ...
5515	PPP2CA	protein phosphatase 2 catalytic s...
1058	CENPA	centromere protein A [Source:HGNC...
991	CDC20	cell division cycle 20 [Source:HG...
25936	NSL1	NSL1 component of MIS12 kinetocho...
1063	CENPF	centromere protein F [Source:HGNC...
348995	NUP43	nucleoporin 43 [Source:HGNC Symbo...
91687	CENPL	centromere protein L [Source:HGNC...
81930	KIF18A	kinesin family member 18A [Source...
11130	ZWINT	ZW10 interacting kinetochore prot...
64105	CENPK	centromere protein K [Source:HGNC...
80776	B9D2	B9 domain containing 2 [Source:HG...
79902	NUP85	nucleoporin 85 [Source:HGNC Symbo...
151648	SGO1	shugoshin 1 [Source:HGNC Symbol;A...
8881	CDC16	cell division cycle 16 [Source:HG...
6249	CLIP1	CAP-Gly domain containing linker ...
55143	CDCA8	cell division cycle associated 8 ...
1783	DYNC1LI2	dynein cytoplasmic 1 light interm...
5519	PPP2R1B	protein phosphatase 2 scaffold su...
57082	KNL1	kinetochore scaffold 1 [Source:HG...
79172	CENPO	centromere protein O [Source:HGNC...
1062	CENPE	centromere protein E [Source:HGNC...
84643	KIF2B	kinesin family member 2B [Source:...
51529	ANAPC11	anaphase promoting complex subuni...
23421	ITGB3BP	integrin subunit beta 3 binding p...
11004	KIF2C	kinesin family member 2C [Source:...
83540	NUF2	NUF2 component of NDC80 kinetocho...
51143	DYNC1LI1	dynein cytoplasmic 1 light interm...
1060	CENPC	centromere protein C [Source:HGNC...
3619	INCENP	inner centromere protein [Source:...
79980	DSN1	DSN1 component of MIS12 kinetocho...
79682	CENPU	centromere protein U [Source:HGNC...
57405	SPC25	SPC25 component of NDC80 kinetoch...
64946	CENPH	centromere protein H [Source:HGNC...
64682	ANAPC1	anaphase promoting complex subuni...
5903	RANBP2	RAN binding protein 2 [Source:HGN...
25909	AHCTF1	AT-hook containing transcription ...
5529	PPP2R5E	protein phosphatase 2 regulatory ...
9184	BUB3	BUB3 mitotic checkpoint protein [...
220134	SKA1	spindle and kinetochore associate...

701	BUB1B	BUB1 mitotic checkpoint serine/th...
6396	SEC13	SEC13 homolog, nuclear pore and C...
1780	DYNC1I1	dynein cytoplasmic 1 intermediate...
57551	TAOK1	TAO kinase 1 [Source:HGNC Symbol;...
11243	PMF1	polyamine modulated factor 1 [Sou...
147841	SPC24	SPC24 component of NDC80 kinetoch...
151246	SGO2	shugoshin 2 [Source:HGNC Symbol;A...
23122	CLASP2	cytoplasmic linker associated pro...
4085	MAD2L1	mitotic arrest deficient 2 like 1...
10393	ANAPC10	anaphase promoting complex subuni...
119504	ANAPC16	anaphase promoting complex subuni...
55839	CENPN	centromere protein N [Source:HGNC...
81565	NDEL1	nudE neurodevelopment protein 1 l...
5347	PLK1	polo like kinase 1 [Source:HGNC S...
79003	MIS12	MIS12 kinetochore complex compone...
699	BUB1	BUB1 mitotic checkpoint serine/th...
7324	UBE2E1	ubiquitin conjugating enzyme E2 E...
55055	ZWILCH	zwilch kinetochore protein [Sourc...
11065	UBE2C	ubiquitin conjugating enzyme E2 C...
9793	CKAP5	cytoskeleton associated protein 5...
378708	CENPS	centromere protein S [Source:HGNC...
29882	ANAPC2	anaphase promoting complex subuni...
246184	CDC26	cell division cycle 26 [Source:HG...
6232	RPS27	ribosomal protein S27 [Source:HGN...
9212	AURKB	aurora kinase B [Source:HGNC Symb...
55920	RCC2	regulator of chromosome condensat...
348235	SKA2	spindle and kinetochore associate...
9735	KNTC1	kinetochore associated 1 [Source:...
5501	PPP1CC	protein phosphatase 1 catalytic s...
54821	ERCC6L	ERCC excision repair 6 like, spin...
401541	CENPP	centromere protein P [Source:HGNC...
51434	ANAPC7	anaphase promoting complex subuni...
1778	DYNC1H1	dynein cytoplasmic 1 heavy chain ...
140735	DYNLL2	dynein light chain LC8-type 2 [So...
54820	NDE1	nudE neurodevelopment protein 1 [...
57405	SPC25	SPC25 component of NDC80 kinetoch...
55920	RCC2	regulator of chromosome condensat...
6790	AURKA	aurora kinase A [Source:HGNC Symbol;Acc:HG...
6795	AURKC	aurora kinase C
891	CCNB1	cyclin B1 [Source:HGNC Symbol;Acc:HGNC:1579]
8555	CDC14B	cell division cycle 14B [Source:HGNC Symbol...
8556	CDC14A	cell division cycle 14A [Source:HGNC Symbol...

983	CDK1	cyclin dependent kinase 1 [Source:HGNC Symb...
201161	CENPV	centromere protein V
9587	MAD2L1BP	MAD2L1 binding protein
84930	MASTL	microtubule associated serine/threonine kinase like
7272	TTK	TTK protein kinase [Source:HGNC Symbol;Acc...
9232	PTTG1	PTTG1 regulator of sister chromatid separat...
4926	NUMA1	nuclear mitotic apparatus protein 1 [Sourc...
51343	FZR1	fizzy and cell division cycle 20 related 1 ...
4659	PPP1R12A	protein phosphatase 1 regulatory subunit 12A
9700	ESPL1	extra spindle pole bodies like 1, separase...
10274	STAG1	stromal antigen 1 [Source:HGNC Symbol;Acc:H...
10735	STAG2	stromal antigen 2 [Source:HGNC Symbol;Acc:H...
8243	SMC1A	structural maintenance of chromosomes 1A [...
9126	SMC3	structural maintenance of chromosomes 3 [S...
79677	SMC6	structural maintenance of chromosomes 6
22974	TPX2	TPX2 microtubule nucleation factor [Source...
23063	WAPL	WAPL cohesin release factor
4751	NEK2	NIMA related kinase 2 [Source:HGNC Symbol;...
10783	NEK6	NIMA related kinase 6 [Source:HGNC Symbol;...
11064	CNTRL	centriolin
51199	NIN	ninein

Supplementary Table 2. Antibodies.

1. antibody	Cat. No	company
Anti-actin	691001	MP Biomedicals
Anti-Aurora B	ab 45145	abcam
Anti-BubR1	ab172581	abcam
Anti-Cdc20	ab183479	abcam
Anti-Cyclin B1	ab181593	abcam
Anti-GAPDH (6C5)	sc-32233	Santa Cruz Biotechnology
Anti-MAD1	ab175245	abcam
Anti-MAD2xl	SA0503	Ag Antonin
Anti-TTK/Mps1	A01132-1	Antibody Picoband
Anti-p53 (1C12)	#2524	Cellsignalling
Anti-phospho-BRCA1 (Ser1524)	#9009	Cell signalling
Anti-phospho-H2A.X (Ser193)	05-636	Millipore
Anti-PLK1	05-844	Millipore
Anti-PP2A-Ca/b	sc-56950	Santa Cruz Biotechnology

2. antibody	Cat. No	company
Goat anti-Human IgG (H+L) Cross-Adsorbed Secondary Antibody	#A-21244	Invitrogen
Goat Anti-Mouse IgG, H&L Chain Specific Peroxidase Conjugate	401215	Sigma-Aldrich
Goat anti-Mouse IgG (H+L) Cross-Adsorbed Secondary Antibody	#A-11001	Invitrogen
Goat Anti-Rabbit IgG, H & L Chain Specific Peroxidase Conjugate	401353	Sigma-Aldrich
Goat anti-Rabbit IgG (H+L) Cross-Adsorbed Secondary Antibody	#A-11008	Invitrogen

Supplementary table 3. Negative GEX z-score genes of PMF/SMF/PV1/11/9 cohort
The table shows the genes negatively downregulated in the PMF/SMF/PV1/11/9 cohort from the upper part of the heatmap in Fig. 7 and Fig. S5.

NCBI (Entrez) Gene Id	Gene Symbol	Gene Description
27338	UBE2S	ubiquitin conjugating enzyme E2 S...
332	BIRC5	baculoviral IAP repeat containing...
64946	CENPH	centromere protein H [Source:HGNC...
91687	CENPL	centromere protein L [Source:HGNC...
147841	SPC24	SPC24 component of NDC80 kinetoch...
79980	DSN1	DSN1 component of MIS12 kinetocho...
81930	KIF18A	kinesin family member 18A [Source...
699	BUB1	BUB1 mitotic checkpoint serine/th...
22974	TPX2	TPX2 microtubule nucleation factor [Source...
57405	SPC25	SPC25 component of NDC80 kinetoch...
9212	AURKB	aurora kinase B [Source:HGNC Symb...
10403	NDC80	NDC80 kinetochore complex compone...
5347	PLK1	polo like kinase 1 [Source:HGNC S...
55143	CDCA8	cell division cycle associated 8 ...
83540	NUF2	NUF2 component of NDC80 kinetocho...
983	CDK1	cyclin dependent kinase 1 [Source:HGNC Symb...
891	CCNB1	cyclin B1 [Source:HGNC Symbol;Acc:HGNC:1579]
1062	CENPE	centromere protein E [Source:HGNC...
7272	TTK	TTK protein kinase [Source:HGNC Symbol;Acc...
220134	SKA1	spindle and kinetochore associate...
9700	ESPL1	extra spindle pole bodies like 1, separase...
3619	INCENP	inner centromere protein [Source:...
4751	NEK2	NIMA related kinase 2 [Source:HGNC Symbol;...
1063	CENPF	centromere protein F [Source:HGNC...
9232	PTTG1	PTTG1 regulator of sister chromatid separat...
11130	ZWINT	ZW10 interacting kinetochore prot...
4085	MAD2L1	mitotic arrest deficient 2 like 1...
55839	CENPN	centromere protein N [Source:HGNC...
79019	CENPM	centromere protein M [Source:HGNC...
84930	MASTL	microtubule associated serine/threonine kinase like
2491	CENPI	centromere protein I [Source:HGNC...
79172	CENPO	centromere protein O [Source:HGNC...

3796	KIF2A	kinesin family member 2A [Source:...
55166	CENPQ	centromere protein Q [Source:HGNC...
11065	UBE2C	ubiquitin conjugating enzyme E2 C...
991	CDC20	cell division cycle 20 [Source:HG...
1058	CENPA	centromere protein A [Source:HGNC...
6790	AURKA	aurora kinase A [Source:HGNC Symbol;Acc:HG...
54908	SPDL1	spindle apparatus coiled-coil pro...
23421	ITGB3BP	integrin subunit beta 3 binding p...
9735	KNTC1	kinetochore associated 1 [Source:...
5905	RANGAP1	Ran GTPase activating protein 1 [...
8243	SMC1A	structural maintenance of chromosomes 1A [...
57122	NUP107	nucleoporin 107 [Source:HGNC Symb...
79682	CENPU	centromere protein U [Source:HGNC...
64105	CENPK	centromere protein K [Source:HGNC...
401541	CENPP	centromere protein P [Source:HGNC...
